# Doxorubicin induces apoptosis by targeting Madcam1 and AKT and inhibiting protein translation initiation in hepatocellular carcinoma cells

**DOI:** 10.18632/oncotarget.4373

**Published:** 2015-06-08

**Authors:** Jiayi Wang, Lifang Ma, Xun Tang, Xiao Zhang, Yongxia Qiao, Yuling Shi, Yanfeng Xu, Zhongyong Wang, Yongchun Yu, Fenyong Sun

**Affiliations:** ^1^ Department of Clinical Laboratory Medicine, Shanghai Tenth People's Hospital of Tongji University, Shanghai, China; ^2^ Translation Medicine of High Institute, Tongji University, Shanghai, China; ^3^ School of Public Health, Shanghai Jiaotong University School of Medicine, Shanghai, China; ^4^ Department of Dermatology, Shanghai Tenth People's Hospital of Tongji University, Shanghai, China; ^5^ Department of Pharmacy, Shanghai Municipal Hospital of Traditional Chinese Medicine affiliated to Shanghai TCM University, Shanghai, China; ^6^ Medical Examination Centre, The First Affiliated Hospital of Wenzhou Medical University, Wenzhou, Zhejiang Province, China; ^7^ Shanghai Municipal Hospital of Traditional Chinese Medicine affiliated to Shanghai TCM University, Shanghai, China

**Keywords:** eIF4E, protein synthesis, caspase activity, 4EBP1, RNA-IP

## Abstract

Doxorubicin (Doxo) is one of the most widely used chemotherapeutic drugs for patients with hepatocellular carcinoma (HCC). Doxo is a DNA intercalating drug that inhibits topoisomerase II. Thereby Doxo has the ability to block DNA replication and induce apoptosis. However, the other targets and mechanisms through which Doxo induces apoptosis to treat HCC still remain unknown. Here, we identified Mucosal vascular addressin cell adhesion molecule 1 (Madcam1) as a potential Doxo target because Madcam1 overexpression suppressed, while Madcam1 depletion stimulated Doxo-induced apoptosis. Furthermore, we first revealed that Doxo can induce apoptosis by blocking protein translation initiation. In contrast, Madcam1 activated protein translation through an opposite mechanism. We also found de-phosphorylation of AKT may be an important pro-apoptotic event that is triggered by Doxo-induced Madcam1 down-regulation. Finally, we revealed that Madcam1 promoted increased AKT phosphorylation, which is essential for maintaining the sensitivity of HCC cells to Doxo treatment. Taken together, we uncovered a potential mechanism for Doxo-induced apoptosis in HCC treatment through targeting Madcam1 and AKT and blocking protein translation initiation.

## INTRODUCTION

Doxo is one of the most widely used chemotherapeutic drugs for HCC treatment [1-3]. Doxo is a DNA intercalating drug that inhibits topoisomerase II [4-5]. Thereby Doxo has the ability to block DNA replication and induce apoptosis. However, the performance of Doxo may be reduced because both DNA and topoisomerase are primarily located in the cell nuclei, which increases challenges such as poor intracellular drug delivery. Therefore, other potential Doxo targets, especially the proteins located at either the cell membrane or the cytoplasm, need to be urgently revealed. Combined use of specific inhibitor against such Doxo targets with Doxo may enhance the efficacy of Doxo in treating HCC.

Madcam1 was originally identified as an endothelial cell adhesion molecule that directs leukocytes into mucosal and inflamed tissues [6]. Madcam1 is overexpressed in pancreatic tumor and lymphoma [7-9], suggesting that Madcam1 plays a critical role in tumorigenesis. However, no studies have currently focused on the function of Madcam1 in HCC. Whether and how Madcam1 participates in the Doxo-induced apoptosis in HCC cells remains elusive.

To date, several studies have demonstrated that the inhibition or stimulation of growth/survival signaling pathways ultimately leads to Doxo-induced cell death in HCC [10-18]. Among these signaling pathways, AKT signaling has been clearly linked to the pathogenesis of HCC [19-21]. A recent study suggests that the use of AKT inhibitors in combination with Doxo may be an attractive therapeutic regimen for HCC treatment [22]. However, the precise relationship between AKT and Doxo is unknown.

In this study, we identified Madcam1 as a potential Doxo target. Although Madcam1 could be down-regulated by Doxo, Madcam1 had an anti-apoptotic function against Doxo. Furthermore, we determined that Doxo induces apoptosis in HCC cells by inhibiting protein translation initiation. We also revealed a novel auto-regulatory loop between Madcam1 and AKT, which plays important roles in the regulation of apoptosis. Finally, we revealed that Madcam1 promoted increased AKT phosphorylation, which is essential for maintaining the sensitivity of HCC cells to Doxo treatment. Thus, we suggested that the use of a Madcam1 inhibitor could enhance the efficacy of Doxo in HCC treatment.

## RESULTS

### Doxorubicin induced apoptosis and reduced Madcam1 expression in HCC cells

In this study, the HCC cell line Bel-7402 was selected because Bel-7402 cells are sensitive to Doxo [23]. Another HCC cell line, SMMC-7721, that exhibits similar carcinogenic properties as Bel-7402 [23-24], and a hepatocyte line, HL-7702, that shows a significant difference from HCC cells [25], were also included in parallel experiments.

**Figure 1:**
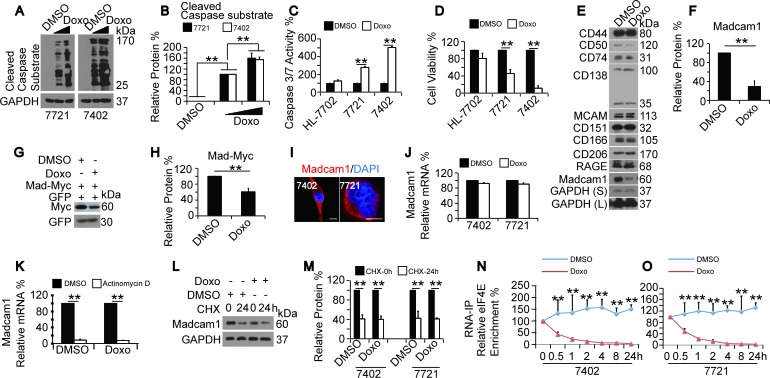
Doxorubicin induced apoptosis and reduced Madcam1 expression **A.-B.** Doxo induced dose-dependent cleavage of Caspase substrates (CCS), as analyzed by WB using an anti-cleaved caspase substrate antibodies, in SMMC-7721 and Bel-7402 cells treated with DMSO or Doxo (final concentration 0.5-2.0 μg/ml) for 24 h. The representative western blots are shown in panel A. The CCS was calculated as the ratio between the levels of cleaved Caspase substrate and GAPDH, and the data are shown in panel B. The groups treated with 0.5 μg/ml Doxo were arbitrarily set to 100%. **C.** Doxo induced Caspase 3/7 activity, as measured by a Caspase 3/7 Glo luciferase kit from Promega, in cells treated with DMSO or Doxo (final concentration 2.0 μg/ml) for 24 h. The “ DMSO” group was arbitrarily set to 100%. **D.** Doxo reduced cell proliferation, as measured by an MTT-based assay, in cells treated with DMSO or Doxo (final concentration 2.0 μg/ml) for 24 h. The “ DMSO” group was arbitrarily set to 100%. **E.** Potential proteins that were regulated by Doxo. Endogenous expression of the indicated proteins were tested by WB in Bel-7402 cells treated with DMSO or Doxo (final concentration 2.0 μg/ml) for 24 h. **F.** The relative levels of Madcam1 protein expression in Bel-7402 cells treated with DMSO or Doxo (final concentration 2.0 μg/ml) for 24 h were calculated as the ratio between Madcam1 and GAPDH. The “DMSO” group was arbitrarily set to 100 %. **G.-H.** Exogenous Madcam1-Myc was measured in Bel-7402 transfected with GFP and Madcam1-Myc expressing plasmids and treated with DMSO or Doxo (final concentration 2.0 μg/ml) for 24 h. Representative WB images of are shown in panel G, the relative expression levels of exogenous Myc were calculated as the ratio to GFP, and the data are shown in panel H. The “DMSO” group was arbitrarily set to 100 %. **I.** Subcellular localization of Madcam1 in Bel-7402 and SMMC-7721 cells, as measured by the immunofluorescence assay. Scale bar, 5 μM. **J.** Madcam1 mRNA levels in Bel-7402 and SMMC-7721 cells treated with DMSO or Doxo (final concentration 2.0 μg/ml) for 24 h, as measured by qPCR assays. The Madcam1 levels were normalized to GAPDH. The “DMSO” group was arbitrarily set to 100 %. **K.** Bel-7402 cells were pretreated with DMSO or Doxo (final concentration 2.0 μg/ml) for 2 h before adding DMSO or actinomycin D (final concentration 10 μg/ml) for further treatment for 24 h. Then, the RNA was extracted and measured by qPCR assays. The Madcam1 levels were normalized to GAPDH. The group treated with DMSO only was arbitrarily set to 100 %. **L.-M.** Doxo had no influence on Madcam1 protein stability. Bel-7402 cells were pretreated with DMSO or Doxo (final concentration 2.0 g/ml) for 2 h before being treated with CHX (final concentration 50 μg/ml) for the indicated times. Then, the cells were harvested for WB using anti-Madcam1 and anti-GAPDH antibodies **L.**. The Madcam1 protein levels were normalized to GAPDH, and the data are shown in panel **M.**. The group treated with CHX at the 0 h point was arbitrarily set to 100 %. **N.-O.** Time-dependent enrichment of eIF4E on the Madcam1 mRNA. Bel-7402 **N.** and SMMC-7721 **O.** cells were treated with same amount of DMSO or Doxo (final concentration 2.0 μg/ml) for the indicated times. Then, the RNA was extracted and subjected into RNA-IP assays using an anti-eIF4E antibody. The “0h” point was arbitrarily set to 100 %. **, Indicates statistical significance between “DMSO” and “Doxo” at specific time points. The data are shown as the means + SD from three independent experiments (including WB). **, *p* < 0.01 using the Student's *t* test.

First, we examined whether Doxo stimulates apoptosis in HCC cells by testing the cleavage of Caspase substrates (CCS) using an anti-cleaved Caspase substrate antibody that recognizes the endogenous levels of Caspase-cleaved proteins with a carboxy-terminal aspartic acid residue. We found that Doxo dose-dependently elevated CCS levels in both SMMC-7721 and Bel-7402 cells (Figure 1A-1B). We also noticed that Doxo treatment led to significantly induced Caspase 3/7 activities in SMMC-7721 and Bel-7402 cells, but not in HL-7702 cells (Figure 1C), suggesting that Doxo can only induce apoptosis in HCC cells but not in normal hepatocytes. Induced apoptosis is usually accompanied by reduced cell proliferation. Therefore, we tested whether Doxo inhibits cell proliferation. We found that compared to cells treated with DMSO, the Doxo-treated SMMC-7721 and Bel-7402 cells had a much lower cell proliferation activity (Figure 1D). In contrast, Doxo was also unable to significantly reduce cell proliferation in HL-7702 cells (Figure 1D).

One purpose of this study is to identify the potential Doxo target(s). Compared to nuclear proteins, cytoplasmic and membrane proteins are much more easily accessed by Doxo in the circulation. Thus, we examined the expression of a series of cancer-related proteins that might bind to the cell membrane before and after Doxo treatment in Bel-7402 cells. We found that Madcam1 was the best candidate that could be down-regulated by Doxo (Figure 1E-1F). In addition to the fact that endogenous Madcam1 could be dose-dependently down-regulated by Doxo in both SMMC-7721 and Bel-7402 cells (Supplementary Figure 1A-1B), exogenous Madcam1-Myc could also be down-regulated by Doxo in Bel-7402 cells (Figure 1G-1H). By adding the protein synthesis inhibitor cycloheximide (CHX) to Bel-7402 cells, Madcam1 was observed as the most unstable protein of the proteins tested (Supplementary Figure 1C). This may explain why Madcam1 had the most rapid response to Doxo treatment.

We then investigated the subcellular localization of Madcam1 in HCC cells. Madcam1 was detected in the cytoplasm but not in the membrane, not only in established HCC cell lines (Figure 1I) but also in HCC tissues (Supplementary Figure 1D), indicating that in addition to its functions as a surface adhesion molecule that mediates cell-to-cell interactions, Madcam1 may have other important functions in HCC cells.

Next, we investigated how Doxo down-regulates Madcam1. We found that Doxo did not significantly change the mRNA levels of Madcam1 in both Bel-7402 and SMMC-7721 cells (Figure 1J). Furthermore, Doxo was unable to accelerate Actinomycin D (a transcription inhibitor)-reduced Madcam1 mRNA levels in Bel-7402 cells (Figure 1K). These results excluded the possibility that Doxo suppresses Madcam1 expression by reducing its transcription and RNA stability. We also found no significant differences in the CHX-reduced Madcam1 to GAPDH protein ratios between DMSO- and Doxo-treated cells (Figure 1L-1M), suggesting that Doxo does not reduce Madcam1 protein stability. Then, we tested whether Doxo suppresses the translation of Madcam1 protein. Eukaryotic initiation factor 4E (eIF4E) plays an important role in protein translation by facilitating the recruitment of other translation factors and the 40S ribosomal subunit to the corresponding mRNAs [26-27]. Using RNA-IP followed by RT-qPCR, we observed a kinetic decrease of eIF4E binding to the Madcam1 mRNA after Doxo was added to both Bel-7402 and SMMC-7721 cells (Figure 1N-1O), demonstrating that Doxo suppresses Madcam1 primarily through the inhibition of eIF4E-mediated protein translation.

### Madcam1 functioned against Doxorubicin in the regulation of apoptosis

**Figure 2:**
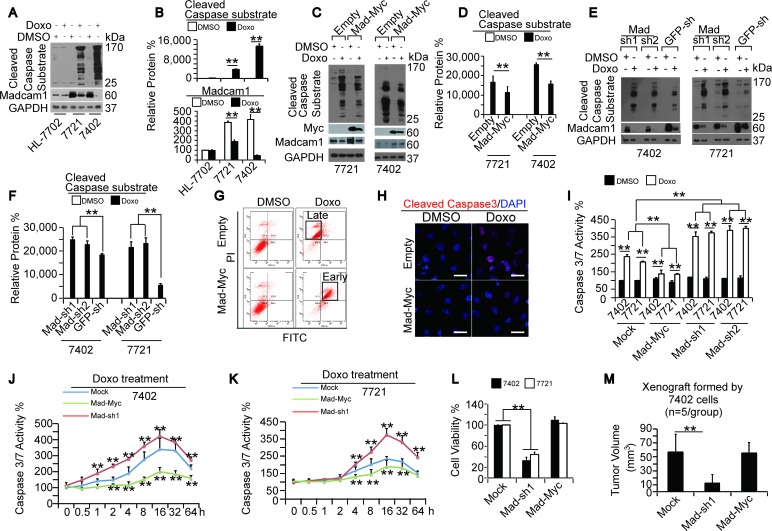
Madcam1 suppressed Doxorubicin-induced apoptosis in HCC cells **A.-B.** CCS and Madcam1 in different cells that were treated with DMSO or Doxo (final concentration 2.0 μg/ml) for 24 h before harvest for WB using an anti-cleaved Caspase substrate antibody. Representative images of the WB are shown in panel A. The levels of CCS and Madcam1 were normalized to GAPDH, and the data are shown in panel B. The data from the “DMSO-treated” HL-7702 cells was arbitrarily set to 100 %. **C.-D.** Madcam1 overexpression reduced Doxo-induced apoptosis. Bel-7402 and SMMC-7721 cells infected with empty or Madcam1-Myc expressing lentiviral plasmids were treated with same amount of DMSO or Doxo (final concentration 2.0 μg/ml) for 24 h before harvest for WB analysis using anti-cleaved Caspase substrate antibodies. Representative images of the WB are shown in panel C. The CCS levels were normalized to GAPDH, and the data are shown in panel D. The SMMC-7721 cells infected with the empty vector and treated with DMSO were arbitrarily set to 100 %. **E.-F.** Madcam1 knockdown reinforced Doxo-induced apoptosis. Control (infected with GFP-sh) and Madcam1 knockdown (infected with Madcam1-sh1 and –sh2, respectively) Bel-7402 or SMMC-7721 cells were treated with DMSO or Doxo (final concentration 2.0 μg/ml) for 24 h before harvest for WB analysis using anti-cleaved Caspase substrate antibodies. Representative images of the WB are shown in panel E. The CCS levels were normalized to GAPDH, and the data are shown in panel F. The cells infected with Mad-sh1 and treated with DMSO were arbitrarily set to 100 %. **G.** Madcam1 prevented Doxo-induced apoptosis, as measured by a flow cytometer using an Annexin V-FITC early apoptosis detection kit, in Bel-7402 cells treated with DMSO or Doxo (final concentration 2.0 μg/ml) for 24 h. **H.** Madcam1 reduced cleaved Caspase 3 expression, as measured by immunofluorescence assay, in Bel-7402 cells treated with DMSO or Doxo (final concentration 2.0 μg/ml) for 24 h. Scale bar, 20 μM. **I.** Madcam1 reduced Doxo-induced and Caspase 3/7 activity. Control (Mock) and Madcam1 overexpressing or knockdown Bel-7402 and SMMC-7721 cells were treated with DMSO or Doxo (final concentration 2.0 μg/ml) for 24 h before harvest for testing the Caspase 3/7 activity using a Caspase 3/7 Glo luciferase kit. Data from the “Mock treated with DMSO” group were arbitrarily set to 100 %. **J.-K.** Time-dependent Caspase 3/7 activities after Doxo (final concentration 0.5 μg/ml) treatment in the indicated Bel-7402 **J.** and SMMC-7721 **K.** cells was measured by a Caspase 3/7 Glo luciferase kit from Promega. The data from the “Mock-0h” group were arbitrarily set to 100 %. **, *p* < 0.01 versus the Mock group at the same time point using the Student's *t* test. **L.** Madcam1 knockdown reduced cell proliferation, as measured by an MTT-based assay, in Bel-7402 and SMMC-7721 cells. The initial cell number is 5,000, and the cells were cultured for another 5 days before the OD value was determined. **M.** Measurement of xenografts generated by Bel-7402 cells *in vivo*. The tumor size formed from the subcutaneous injection of Bel-7402 cells with the different indicated treatments was measured with calipers at 30 days after injection and calculated as 0.5 × L × W^2^(*n* = 5 per group). The data are shown as the means + SD from three independent experiments (including WB). **, *p* < 0.01 using the Student's *t* test.

We described above that the dose-dependent increases in CCS levels were accompanied by dose-dependent decreases in Madcam1 levels in HCC cells treated with increasing concentrations of Doxo (Figure 1A and Supplementary Figure 1A). Here, we compared CCS and Madcam1 levels in different cells with or without Doxo treatment, and found that the most significantly increased CCS levels were accompanied by the most significantly decreased Madcam1 levels in Bel-7402 cells, mildly increased CCS levels were accompanied with mildly decreased Madcam1 levels in SMMC-7721 cells, and there was no decrease of either the CCS or Madcam1 levels in HL-7702 cells (Figure 2A-2B). These results led us to propose that Madcam1 may function against Doxo in the regulation of apoptosis. To address this, we added Doxo into control and Madcam1 overexpressing or knockdown cells. We found that Doxo induced less accumulation of CCS in Madcam1 overexpressing Bel-7402 and SMMC-7721 cells compared to the control (Figure 2C-2D). In contrast, Doxo treatment induced a greater accumulation of CCS in Madcam1 knockdown Bel-7402 and SMMC-7721 cells compared to the control (Figure 2E-2F). However, neither overexpression nor depletion of Madcam1 expression in Doxo-treated or untreated HL-7702 cells significantly changed the CCS levels (Supplementary Figure 2).

Using an early apoptosis detection kit from Cell Signaling Technology LTD (CST), we found that Madcam1 overexpression resulted in delayed Doxo-induced apoptosis (Figure 2G). Furthermore, Doxo was capable of inducing significant cleaved Caspase 3 expression (Figure 2H), and increased levels of cleaved Caspase 3 are regarded as a hallmark of apoptosis [28]. However, the Doxo increase in the cleaved Caspase 3 levels was significantly reduced in Madcam1 overexpressing Bel-7402 cells compared to the control cells (Figure 2H). The data from testing the Caspase 3/7 activities in Madcam1 overexpressing or knockdown Bel-7402 and SMMC-7721 cells were also in accord with these results (Figure 2I). Collectively, we suggested that Madcam1 may protect HCC cells from Doxo-induced apoptosis.

Madcam1 overexpression and knockdown appears to change the course of Doxo-induced apoptosis (Figure 2C-2I). However, it is not known whether Madcam1 changes the time course or overall level of Doxo-induced apoptosis. We found that Doxo treatment significantly increased Caspase 3/7 activity in both Bel-7402 and SMMC-7721 cells in a time-dependent manner, and reached a peak at approximately 16 h, then decreased gradually (Figure 2J-2K). Furthermore, compared to the control, Madcam1 knockdown enhanced, while Madcam1 overexpression attenuated Doxo-induced Caspase 3/7 activities (Figure 2J-2K). Interestingly, the time course of Doxo-induced apoptosis was not changed by either Madcam1 knockdown or overexpression (Figure 2J-2K). These results suggested that Madcam1 could only change the overall level, but not the time course of Doxo-induced apoptosis.

Then, we tested whether changes in Madcam1 leads to functional differences in the HCC cells. We found that Madcam1 knockdown significantly impaired cell proliferation in both Bel-7402 and SMMC-7721 cells, as analyzed by an MTT-based assay (Figure 2L). However, Madcam1 overexpression in HCC cells had no significant influence on cell proliferation (Figure 2L), which may be because endogenous Madcam1 is already overloaded in HCC cells (Figure 7A-7B). Similarly, in xenograft mouse models, we found that only Madcam1 knockdown could lead to impaired tumor growth from Bel-7402 cells *in vivo* (Figure 2M). These results suggested that Madcam1 can not only change the Caspase levels, but it also functional in the maintenance of the transformed phenotype in HCC cells.

### Doxorubicin and Madcam1 controlled protein translation initiation

**Figure 3:**
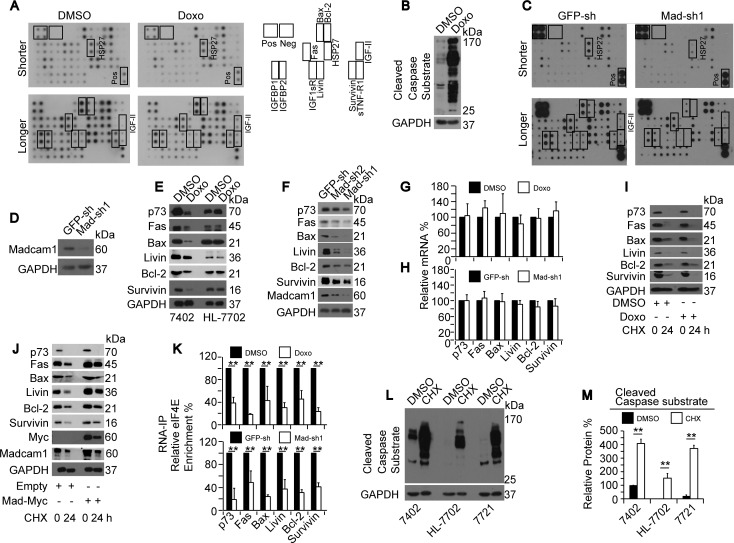
Madcam1 suppressed Doxorubicin in translation initiation **A.** Screening for possible Doxo target proteins, as analyzed by WB using a human apoptosis antibody array (containing 43 Targets) from Abcam. Bel-7402 cells were treated with DMSO or Doxo (final concentration 2.0 μg/ml) for 24 h before being subjected to the array analysis. The images of the array membrane are shown on the left. Shorter, shorter exposure; Longer, longer exposure. The spot positions of protein on the membrane are listed on the right. **B.** The same samples from panel A were subjected to WB for the confirmation of Doxo-induced apoptosis using anti-cleaved Caspase substrate antibodies. **C.** Screening targets before and after Madcam1 knockdown. Bel-7402 cells infected with GFP-sh or Madcam1-sh1 were harvested for WB using the array described in panel A. Shorter, shorter exposure; Longer, longer exposure. **D.** Madcam1-sh1knockdown efficiency in the samples from panel C. **E.** Representative western blots for the indicated proteins in Bel-7402 and HL-7702 cells treated with DMSO or Doxo (final concentration 2.0 μg/ml) for 24 h. **F.** Representative western blots of the indicated proteins in Bel-7402 cells infected with GFP-sh, Madcam1-sh1 or Madcam1–sh2. **G.-H.** mRNA levels in Bel-7402 cells treated with DMSO or Doxo (final concentration 2.0 μg/ml) for 24 h **G.** or in Bel-7402 cells infected with GFP-sh or Madcam1-sh1 **H.**. The mRNA levels were measured by qPCR assays. The levels in the “DMSO” or “GFP-sh” groups were arbitrarily set to 100 %. **I.** Doxo had no impact on protein stability. Bel-7402 cells were pretreated with DMSO or Doxo (final concentration 2.0 μg/ml) for 2 h before adding CHX for the indicated times. Then, the cell lysates were prepared for WB. **J.** Madcam1 had no effect on protein stability. Bel-7402 cells stably transfected with the empty or Madcam1-Myc expressing plasmids were treated with CHX for the indicated times. Then, the cell lysates were prepared for WB. **K.** Doxo and Madcam1 had opposite influences on eIF4E binding. The RNA was extracted from Bel-7402 cells treated with DMSO or Doxo (final concentration 2.0 μg/ml) for 4 h (upper panel) or Bel-7402 cells infected with GFP-sh or Madcam1-sh1 (lower panel), and subjected to RNA-IP assays using anti-eIF4E antibodies. The data from the “DMSO” or “GFP-sh” groups were arbitrarily set to 100 %. **L.-M.** Inhibiting protein synthesis induced apoptosis. Bel-7402, HL-7702 and SMMC-7721 cells were treated with DMSO or CHX (final concentration 50 μg/ml) for 24 h before harvest for WB using anti-cleaved Caspase substrate antibodies. Representative images of the WB are shown in panel L. The relative CCS levels were calculated as the ratio to GAPDH, and the data are shown in the panel M. The data are shown as the means + SD from three independent experiments. **, *p* < 0.01 using the Student's *t* test.

Then, we investigated the potential mechanisms by which Doxo regulates apoptosis in HCC cells by screening targets using a human apoptosis antibody array from Abcam. As shown in Figure 3A, the density of the signals for the positive control were similar between samples, suggesting that the cell lysates from DMSO- or Doxo-treated Bel-7402 cells were loaded equally onto the arrays. We were confident with the array data because the spot signals for three proteins, HSP27, IGF-II and sTNF-R1, were stronger in the Doxo-treated samples compared to the DMSO-treated control (Figure 3A). These results were consistent with the observations from other studies that described that these three proteins can be induced by Doxo [29-31]. Furthermore, these same Doxo-treated samples that were loaded onto the array showed significantly induced CCS levels (Figure 3B), further demonstrating the high quality of the samples. Interestingly, the signals representing the other apoptosis-related proteins were much weaker in the Doxo-treated samples compared to the DMSO-treated control (Figure 3A), suggesting that Doxo may control protein expression through a common mechanism.

Because Madcam1 had opposite functions in the regulation of apoptosis, we hypothesized that Madcam1knockdown has similar results to Doxo treatment. As expected, Madcam1knockdown led to a decrease of almost all of the tested target proteins, including Madcam1 itself (Figure 3C-3D), indicating the important role of Madcam1 in maintaining protein homeostasis. To verify the results from the arrays, we re-examined three pro-apoptotic proteins, including Fas, Bax and p73, and three anti-apoptotic proteins, including Livin, Bcl-2 and Survivin, before and after the treatments listed above. Among these six proteins, five proteins were already included in the array, and p73 was added to avoid systematic error generated by the array-based analysis. By performing the experiments in Bel-7402 and HL-7702 cells, we found that Doxo treatment led to a significant decrease in protein expression only in Bel-7402 cells, but not in HL-7702 cells (Figure 3E and Supplementary Figure 3A), suggesting that Doxo only reduces protein expression in HCC cells but not in hepatocytes. In Madcam1 depleted Bel-7402 cells, down-regulation of protein expression was also observed (Figure 3F and Supplementary Figure 3B). The above results suggested a negative correlation between Doxo and Madcam1 in the regulation of protein expression.

Next, we investigated how Doxo and Madcam1 regulate protein expression. First, we examined the corresponding mRNA levels of these six proteins by qPCR, and found that the mRNA levels were not reduced by either Doxo treatment or Madcam1 knockdown (Figure 3G-3H), suggesting both Doxo and Madcam1 did not influence transcription or RNA stability. We further excluded the possibility that Doxo or Madcam1 suppresses or stimulates protein stability because the reduced protein ratios at the beginning and end of CHX treatment were similar between paired DMSO- and Doxo-treated Bel-7402 cells, as well as paired control and Madcam1 overexpressing Bel-7402 cells (Figure 3I-3J and Supplementary Figure 3C-3D). The results obtained from these six proteins were quite similar with those from Madcam1; therefore, we tested whether Doxo or Madcam1 impact the translation of these proteins. As expected, we found that eIF4E enrichment to these mRNAs was significantly reduced in Doxo-treated or Madcam1 knockdown Bel-7402 cells (Figure 3K), suggesting that both Doxo and Madcam1 influence eIF4E-mediated translation initiation. Then, we tried to assemble the conditions under which protein translation is inhibited using a protein synthesis inhibitor, CHX. We found that CHX induced the CCS level increased more significantly than other drugs, including Doxo, CHX, Actinomycin D (transcription inhibitor) and MG132 (proteasome inhibitor) (Supplementary Figure 3E-3F). Furthermore, CHX-induced CCS levels were detected in Bel-7402, SMMC-7721 and HL-7702 cells (Figure 3L-3M), suggesting that inhibiting protein synthesis can also lead to apoptosis.

### Doxorubicin suppressed, while Madcam1 stimulated eIF4E activation

**Figure 4:**
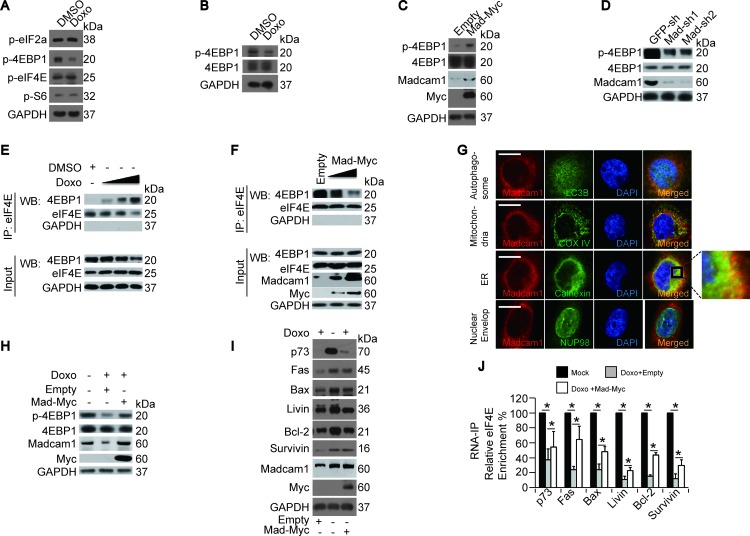
Doxorubicin and Madcam1 had opposite effects on eIF4E **A.** Doxo reduced 4EBP1 phosphorylation. Phosphorylation of translation-associated proteins was detected in Bel-7402 cells treated with DMSO or Doxo (final concentration 2.0 μg/ml) for 4 h before harvest for WB using the indicated antibodies. **B.** p-4EBP1 and 4EBP1 were detected by WB in Bel-7402 cells treated with DMSO or Doxo (final concentration 2.0 μg/ml) for 4 h before harvest. **C.** Madcam1 overexpression induced 4EBP1 phosphorylation, as detected by WB, in Bel-7402 cells transfected with empty or Madcam1-Myc expressing plasmids. **D.** Madcam1 knockdown reduced 4EBP1 phosphorylation, as detected by WB, in Bel-7402 cells infected with GFP-sh, Madcam1-sh1 or Madcam1–sh2. **E.** Doxo induced 4EBP1 binding to eIF4E. Bel-7402 cells were treated with DMSO or increasing concentrations of Doxo (final concentration from 0.5-2.5 μg/ml) for 5 h before harvest in IP lysis buffer. Endogenous eIF4E was then immunoprecipitated with an anti-eIF4E antibody and 4EBP1 co-immunoprecipitation was detected by WB. **F.** Madcam1 reduced 4EBP1 binding to eIF4E. Endogenous eIF4E was immunoprecipitated with an anti-eIF4E antibody and 4EBP1 co-immunoprecipitation was detected by WB for control (transfected with Empty vector) and Madcam1-Myc transfected Bel-7402 cells, using increasing amounts of ectopically expressed Madcam1-Myc. **G.** Identification of the subcellular localization of Madcam1. Co-localization of Madcam1 and possible organelles was analyzed in Bel-7402 cells by immunofluorescence assays using a combination of anti-Madcam1 (red) and anti-LC3B (autophagosome marker, green), anti-COX IV (mitochondria marker, green), anti-Calnexin (endoplasmic reticulum (ER) marker, green) or anti-NUP98 (nuclear envelop marker, green) antibodies. The area indicated by the square was enlarged on the right side. Scale bar, 10 μM. **H.** Madcam1 reversed Doxo-induced dephosphorylation of 4EBP1. Bel-7402 cells were treated with or without Doxo (final concentration 2.0 μg/ml) for 4 h in the presence or absence of ectopically expressed Madcam1-Myc before harvest for WB using anti-p-4EBP1 and 4EBP1 antibodies. **I.** Madcam1 prevented Doxo-induced protein down-regulation. Bel-7402 cells were treated as described in Figure 4H and were harvested for WB using the indicated antibodies. **J.** Madcam1 inhibited Doxo-induced dissociation of eIF4E from the mRNA. The RNA was extracted from Bel-7402 cells that were treated as in Figure 4h for RNA-IP assays using anti-eIF4E antibodies. The data from the “Mock” group were arbitrarily set to 100 %. The data are shown as the means + SD from three independent experiments (including WB). *, *p* < 0.05 using the Student's *t* test.

To further understand the mechanism by which Doxo reduces protein translation initiation, we screened possible target(s) that may influence translation using a translational control antibody screening kit from CST. We found that Doxo treatment only resulted in a decrease in the p-4EBP1 level, but had no significant effects on the phosphorylation of other proteins (Figure 4A). To exclude the possibility that the down-regulation of p-4EBP1 was due to the down-regulation of total 4EBP1, we re-examined p-4EBP1 and total 4EBP1 in DMSO- or Doxo-treated Bel-7402 cells, and found that only p-4EBP1 was down-regulated, while total 4EBP1 had no obvious changes (Figure 4B and Supplementary Figure 4A). Then, we tested whether Madcam1 stimulates 4EBP1 phosphorylation and observed an up-regulation of the p-4EBP1/total-4EBP1 ratio in Madcam1 overexpressing Bel-7402 cells (Figure 4C and Supplementary Figure 4B), while the p-4EBP1/total-4EBP1 ratio was down-regulated in Madcam1 knockdown Bel-7402 cells (Figure 4D and Supplementary Figure 4C).

It has been reported that hyperphosphorylated 4EBP1 prevents it from binding to and inhibiting eIF4E [32]. Therefore, we hypothesized that Doxo and Madcam1 may have opposite roles in the interaction between 4EBP1 and eIF4E. To address this, we performed co-IP assays and found that the Doxo dose-dependently increased the 4EBP1 levels in the immunoprecipitations with anti-eIF4E antibodies (Figure 4E), suggesting that Doxo stimulates 4EBP1 binding to eIF4E. In contrast, Madcam1 overexpression resulted in a decreased concentration of 4EBP1 in protein complexes immunoprecipitated by anti-eIF4E antibodies (Figure 4F), indicating that Madcam1 facilitates the release of eIF4E from the eIF4E-4EBP1 complexes.

Then, we investigated the subcellular organelle localization of Madcam1 by confocal microscopy using a combination of anti-Madcam1 antibodies and antibodies against protein markers of different organelles. We found that Madcam1 was significantly co-localized with Calnexin, a marker of the endoplasmic reticulum (ER), in the cytoplasm (Figure 4G). Because the ER interacts directly with the ribosomes, which are intracellular protein factories [33], the overlap of Madcam1 with ER further indicates that Madcam1 may stimulate protein synthesis. However, we did not detect any obvious co-localization signals with LC3B (autophagosome marker), COXIV (mitochondria marker) and NUP98 (nuclear envelop marker) (Figure 4G), suggesting that Madcam1 does not functions in autophagy or mitochondria-mediated apoptosis.

Next, we tried to determine whether Madcam1 is a direct target of Doxo in its regulation of protein translation. We found that Madcam1 overexpression could partially reverse the Doxo-induced de-phosphorylation of 4EBP1 (Figure 4H and Supplementary Figure 4D), down-regulation of protein expression (Figure 4I and Supplementary Figure 4E) and dissociation of eIF4E from the target mRNAs (Figure 4J), suggesting that Doxo-induced apoptosis and Doxo-impaired protein translation may, at least in part, occur by targeting Madcam1.

### AKT acted as a common target of Doxorubicin and Madcam1

**Figure 5:**
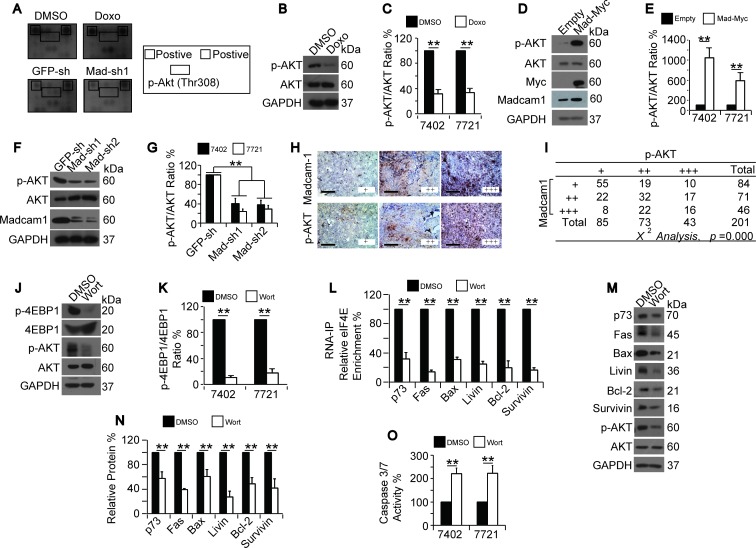
Doxorubicin inhibited while Madcam1 stimulated AKT **A.** Screening for possible signaling nodes that were regulated by both Doxo and Madcam1. Bel-7402 cells treated with DMSO or Doxo (final concentration 2.0 μg/ml) for 4 h as well as Bel-7402 cells infected with GFP-sh or Madcam1-sh1 were harvested and subjected to the Pathscan™ antibody array (CST). The array images are shown on the left side, and the spots representing the positive control and p-AKT (Thr308) are indicated on the right side by the square and rectangle, respectively. **B.-C.** Verification of Doxo-induced AKT de-phosphorylation. Western blots of p-AKT and AKT from Bel-7402 cells treated with DMSO or Doxo (final concentration 2.0 μg/ml) for 4 h **B.**. The levels of p-AKT in Bel-7402 and SMMC-7721 cells were normalized to AKT **C.**. The data from the “DMSO” group were arbitrarily set to 100%. **D.-E.** Madcam1 overexpression induced AKT phosphorylation. Representative western blots of p-AKT and AKT from Bel-7402 cells transfected with the empty or Madcam1-Myc expressing plasmids **D.**. The p-AKT levels from Bel-7402 and SMMC-7721 cells were normalized to f AKT, and the data from the “Empty” group were arbitrarily set to 100% **E..F.-G.** Madcam1 knockdown reduced AKT phosphorylation. Representative western blots of p-AKT and AKT from Bel-7402 cells infected with GFP-sh, Madcam1-sh1 or Madcam1–sh2 **F.**. The p-AKT levels from Bel-7402 and SMMC-7721 cells were normalized to AKT, and the data from the “GFP-sh” group were arbitrarily set to 100% **G.**.**H.-I.** Representative images of TMA stained with anti-Madcam1 or anti-p-AKT antibodies. Scale bar, 200 M **H.**. The TMA data were analyzed using the χ^2^ test **I.**. **J.-K.** Blockage of PI3K/AKT inhibited 4EBP1 phosphorylation. Representative western blots of the indicated proteins from Bel-7402 cells treated with DMSO or Wortmannin (final concentration 50 μM) for 4 h **J.**. The ratio between p-4EBP1 and 4EBP1 are shown in panel **K.**. The data from the “DMSO” group were arbitrarily set to 100%. **L.** Blockage of PI3K/AKT inhibited eIF4E binding to the mRNA, as measured by RNA-IP followed by qPCR using anti-eIF4E antibodies and the indicated primer sets, in Bel-7402 cells treated with DMSO or Wortmannin (final concentration 50 μM) for 4 h. The data from the “DMSO” group were arbitrarily set to 100%. **M.-N.** Blockage of PI3K/AKT reduced protein expression. Western blots of the indicated proteins from Bel-7402 cells treated with same amount of DMSO or Wortmannin (final concentration 50 μM) for 24 h. Representative images of the WB are shown in panel M. The relative levels of the indicated proteins were calculated as the ratio to GAPDH, and the data are shown in panel N. The data from the “DMSO” group were arbitrarily set to 100%. **O.** Blockage of PI3K/AKT induced apoptosis, as measured by a Caspase 3/7 Glo-luciferase kit, in Bel-7402 and SMMC-7721 cells treated with DMSO or Wortmannin (final concentration 50 μM) for 24 h. The data are shown as the means + SD from three independent experiments (including WB). **, *p* < 0.01 using the Student's *t* test.

To enhance the efficacy of Doxo-mediated apoptosis in HCC cells, it is important to identify the signaling cascade(s) involved. Using a Pathscan™ apoptosis and stress signaling array from CST, we found that AKT phosphorylation at Thr308 was a potential target because both Doxo treatment and Madcam1 knockdown in Bel-7402 cells resulted in decreased phosphorylation at this site (Figure 5A). By repeating these experiments in Bel-7402 and SMMC-7721 cells, we demonstrated that Doxo treatment only decreased the phosphorylated form of AKT (p-Thr308-AKT, hereafter p-AKT) and was unable to decrease total AKT (Figure 5B-5C). In contrast, Madcam1 overexpression led to increased p-AKT levels (Figure 5D-5E), while Madcam1 depletion with two independent shRNAs led to decreased p-AKT levels (Figure 5F-5G). Tissue microarray analysis (TMA) also demonstrated a close correlation between p-AKT and Madcam1 in HCC tissues (χ^2^ test, *p* = 0.000, Figure 5H-5I). These results suggested that Madcam1 down-regulation may be an intermediate step during Doxo-induced reductions in p-AKT levels.

We described above that Doxo and Madcam1 regulate protein translation by controlling 4EBP1 phosphorylation. Here, we tested whether AKT also increases 4EBP1 phosphorylation. By inhibiting PI3K/AKT signaling with Wortmannin, a PI3K/AKT inhibitor, we found that blocking PI3K/AKT decreased the p-4EBP1 levels (Figure 5J-5K), which was similar to the results from Doxo treatment or Madacam1 knockdown. We also observed that Wortmannin treatment led to the dissociation of eIF4E from the target mRNAs (Figure 5L), the subsequent down-regulation of protein expression (Figure 5M-5N), and, finally, the remarkable induction of apoptosis (Figure 5O). Because AKT inhibition resembled Doxo treatment and Madcam1 knockdown, we suggested that AKT may act as a common target of Doxo and Madcam1.

Interestingly, we found that AKT inhibition by either Wortmannin or AKT-shRNA led to a decrease in Madcam1 (Supplementary Figure 5A-5B), suggesting that AKT also has a positive feedback on Madcam1 regulation.

### Interaction between Madcam1 and AKT in the regulation of apoptosis

**Figure 6:**
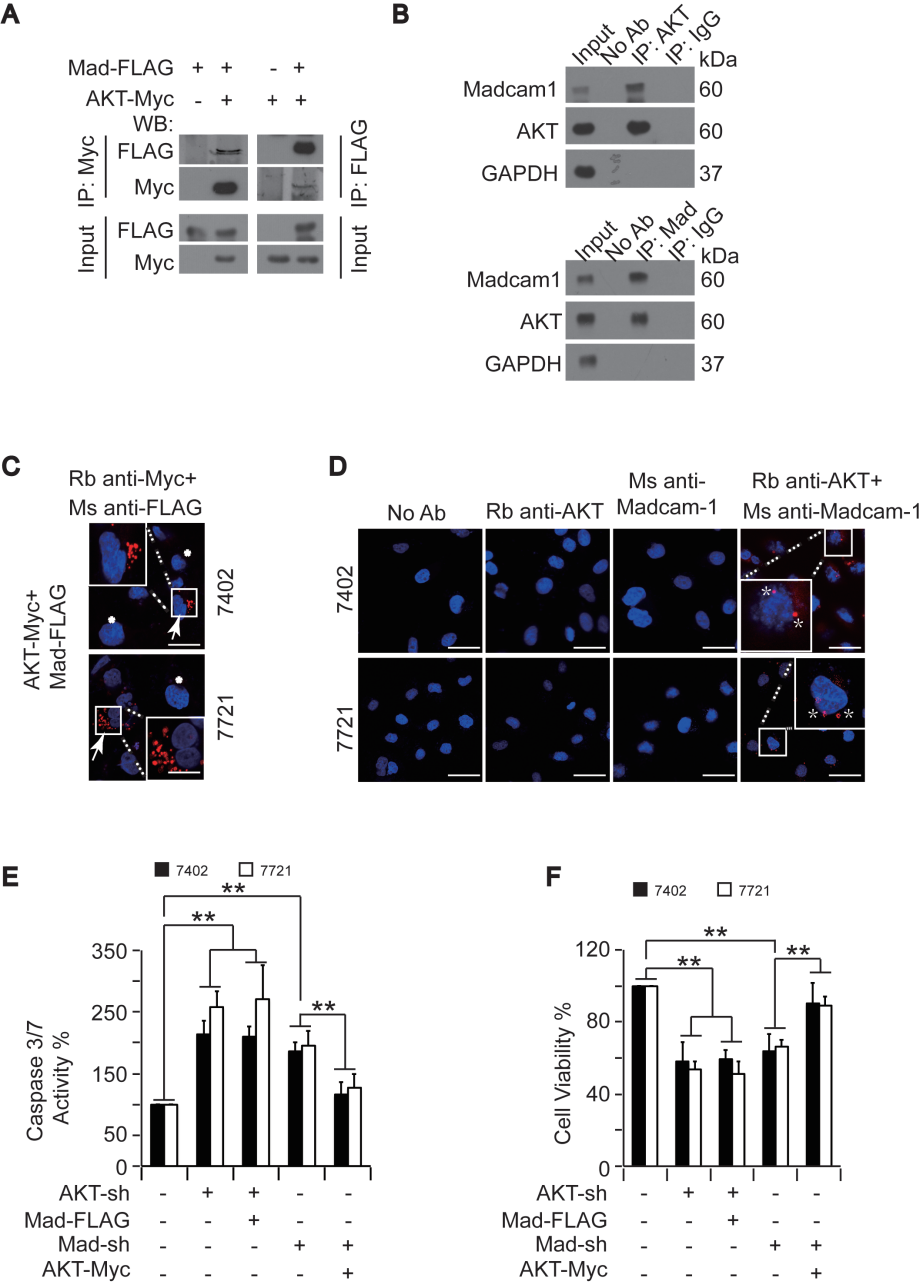
The interaction between Madcam1 and AKT was critical for the regulation of apoptosis **A.** Madcam1 bound AKT. Madcam1-FLAG was co-transfected with AKT-Myc into Bel-7402 cells, as indicated. Madcam1 and AKT associations were examined by reciprocal co-IP, as indicated. **B.** The interaction between endogenous AKT and Madcam1 was measured by reciprocal co-IP using the indicated antibodies. A control immunoglobulin G (IgG) was used as the negative control for IP. **C.** Red Fluorescent emissions in Bel-7402 and SMMC-7721 cells co-transfected with exogenous AKT-Myc and Madcam1-FLAG were detected by a Duolink™ proximity ligation (PLA) kit. The areas with red signals were indicated and enlarged by a square. Scale bar, 20 μM. The arrow indicates cells co-transfected with AKT-Myc and Madcam1-FLAG, while the asterisk indicates cells without successful transfection or with transfection of either AKT-Myc or Madcam1-FLAG. **D.** The interactions between endogenous AKT and Madcam1 in Bel-7402 and SMMC-7721 cells were detected by a Duolink™ proximity ligation (PLA) kit using anti-AKT and anti-Madcam1 antibodies, as indicated. Parallel negative controls were also performed using no antibody (Ab) or only one antibody as indicated. The areas with red signals were indicated and enlarged by a square. Scale bar, 100 μM. The asterisk indicates strong interaction signals. Ms, mouse origin; Rb, rabbit origin. **E.-F.** AKT acted as a downstream effecter of Madcam1. Apoptosis was evaluated by a Caspase 3/7 Glo-luciferase kit **E.** in Bel-7402 and SMMC-7721 cells with or without AKT or Madcam1 knockdown in the presence or absence of ectopically expressed Madcam1-FLAG or AKT-Myc. Cell proliferation was measured by an MTT-based assay **F.**. The data from cells without any treatment were arbitrarily set to 100 %. The data are shown as the means + SD from three independent experiments. **, *p* < 0.01 using the Student's *t* test.

To confirm the interaction between Madcam1 and AKT, we performed reciprocal co-IP experiments in Bel-7402 cells and found that exogenous Madcam1-FLAG could be readily pulled down by AKT-Myc and vice versa (Figure 6A). Co-IPs for endogenous AKT and Madcam1 proteins in Bel-7402 cells also demonstrated that these two proteins readily co-immunoprecipitated (Figure 6B).

However, co-immunoprecipitation does not necessarily mean there is a physical interaction between proteins [34]. Thus, the potential interaction between Madcam1 and AKT was further evaluated using the Duolink™ in situ proximity ligation assay (PLA) kit and confocal laser scanning microscopy. Here, the appearance of red fluorescent emission indicates a positive interaction between Madcam1 and AKT. We only found discrete bright red fluorescent emissions in the Bel-7402 and SMMC-7721 cells that were co-transfected with exogenous AKT-Myc and Madcam1-FLAG, but not in the cells without successful transfection of both of the proteins (Figure 6C). Furthermore, red fluorescent emissions were also detected in Bel-7402 and SMMC-7721 cells co-incubated with both anti-Madcam1 and anti-AKT antibodies (Figure 6D), suggesting endogenous Madcam1 and AKT undergo protein–protein interactions. However, red fluorescent emissions were not observed in the negative controls, which were incubated without antibodies (No Ab) or excluded one of the primary antibodies, either anti-AKT or anti-Madcam1, further validating our conclusion.

Then, we investigated the role of the AKT and Madcam1 interaction in the regulation of apoptosis and cell proliferation. We found that knocking down either AKT or Madcam1 resulted in significantly increased Caspase 3/7 activities in both Bel-7402 and SMMC-7721 cells (Figure 6E). However, only the Madcam1 knockdown increases in the Caspase 3/7 activities could be reduced by the simultaneous overexpression of AKT, while overexpression of Madcam1 was unable to reduce the AKT knockdown increases in the Caspase 3/7 activities (Figure 6E). Similarly, co-overexpression of AKT could rescue impaired cell proliferation in Madcam1-depleted Bel-7402 and SMMC-7721 cells, whereas co-overexpression of Madcam1 was unable to reverse impaired cell proliferation in AKT-depleted cells (Figure 6F). These results suggested that AKT may act as a downstream effector of Madcam1 in the regulation of apoptosis and cell proliferation.

### Doxorubicin sensitivity relied on the p-AKT level

**Figure 7:**
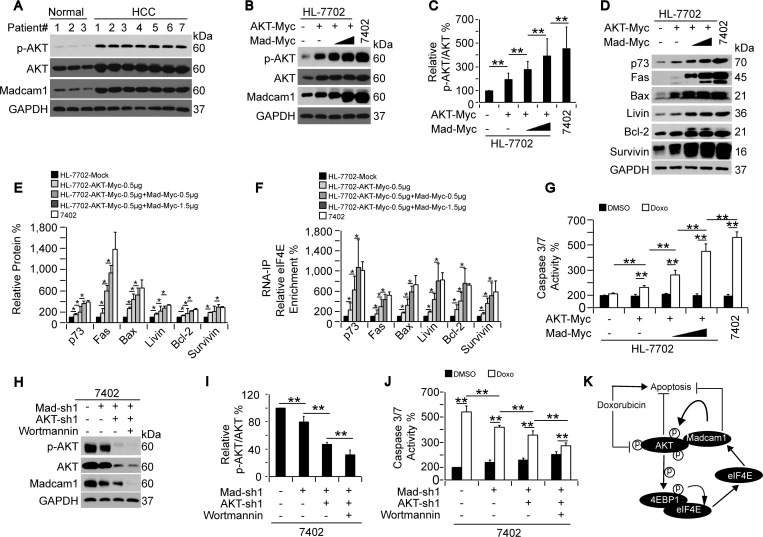
Doxorubicin sensitivity depended on the p-AKT level **A.** Western blots of p-AKT, AKT and Madcam1 in HCC tissues and adjacent normal liver tissues. Normal samples #1, 2, and 3 were paired with the HCC samples #1, 2, and 3, while HCC samples #4, 5, 6, and 7 had no paired normal samples. **B.-C.** Sequential overexpression of AKT and Madcam1 increased AKT phosphorylation, as measured by WB, in HL-7702 cells transfected with AKT-Myc and increasing concentrations (0.5-1.5 μg) of Madcam1-Myc expressing plasmids. Endogenous protein expression in Bel-7402 cells was also examined **B.**. The data were shown as the p-AKT to AKT ratio in panel **C.**, and the data from the “Mock (without treatment)” group were arbitrarily set to 100 %. **D.-E.** Sequential overexpression of AKT and Madcam1 increased protein expression. Western blots of the indicated proteins from HL-7702 cells with the same treatment as in Figures 7B and 7C. Protein expression in the Bel-7402 cells served as the control. Representative images of the WB are shown in panel D. The relative levels of the indicated proteins were calculated as the ratio to GAPDH, and the data are shown in panel E. The data from the “HL-7702 Mock (without treatment)” group were arbitrarily set to 100%. **F.** Sequential overexpression of AKT and Madcam1 recruited eIF4E binding to the mRNAs, as measured by RNA-IP followed by RT-qPCR using anti-eiF4E antibodies and the indicated primer sets, in HL-7702 cells with the same treatment as in Figure 7B-7C. Bel-7402 cells served as the control. The data from the “HL-7702 Mock (without treatment)” group were arbitrarily set to 100 %. **G.** Sequential overexpression of AKT and Madcam1 increased Doxo sensitivity. HL-7702 cells with the same treatment as in Figure 7B-7C were treated with DMSO or Doxo (final concentration 2.0 μg/ml) for 24 h before harvest for Caspase 3/7 activity measurements using a Caspase Glo-luciferase kit. Bel-7402 cells served as the control. The data from the “HL-7702 Mock-DMSO” group were arbitrarily set to 100 %. **H.-I.** Sequential knockdown of Madcam1 and AKT reduced AKT phosphorylation. Bel-7402 cells infected with Madcam-sh1 and AKT-sh1 and treated with or without Wortmannin (final concentration 50 μM) for 24 h were subjected to WB to detect p-AKT, AKT and Madcam1 **H.**. The data were shown as the p-AKT to AKT ratio in panel **I.**, and the data from the untreated cells were arbitrarily set to 100 %. **J.** Sequential knockdown of Madcam1 and AKT reduced Doxo-induced Caspase 3/7 activity, as measured by a Caspase 3/7 Glo-luciferase kit, in Bel-7402 cells with the same treatment as in Figure 7G-7H. The data from the control DMSO-treated cells were arbitrarily set to 100 %. **K.** Illustration of a possible mechanism underlying the critical interaction between Madcam1 and AKT in the Doxorubicin-mediated regulation of apoptosis in HCC cells. The data are shown as the means + SD from three independent experiments (including WB, except panel A). *, *p* < 0.05 and **, *p* < 0.01 using the Student's *t* test.

On the basis of the data described above, we hypothesized that the HCC-specific increased sensitivity to Doxo may rely on the Madcam1-mediated increased levels of p-AKT. To address this, we first examined endogenous p-AKT in HCC and adjacent normal liver tissues, and found both p-AKT and AKT were up-regulated in the HCC tissues compared to the normal liver (Figure 7A). However, we did not believe that the high p-AKT expression levels were due to the high total AKT expression levels because the ratios of AKT/GAPDH were up-regulated 2- to 3-fold, while the ratios of p-AKT/AKT were up-regulated more obviously (3- to 5-fold) in the HCC tissues compared to the normal liver tissues (Supplementary Figure 6A-6B). As expected, Madcam1 was also elevated in the HCC tissues compared to the normal liver tissues (Figure 7A and Supplementary Figure 1D and 6C), suggesting that the up-regulated Madcam1 levels may be another factor, in addition to the elevations in endogenous AKT levels, that stimulates p-AKT *in vivo*.

To simulate the HCC-like, increased p-AKT levels in hepatocyte, we consequently overexpressed exogenous AKT-Myc and Madcam1-Myc in HL-7702 cells, and noticed a gradual up-regulation of the p-AKT levels (Figure 7B-7C). This was accompanied by gradually induced protein expression (Figure 7D-7E) and enhanced eIF4E recruitment to the mRNAs (Figure 7F), which ultimately reached levels resembling the Bel-7402 cells (Figure 7B-7F). Then, we examined Doxo-induced Caspase 3/7 activities in HL-7702 cells with different p-AKT levels, as indicted in Figure 7B-7C, and found that the extent of Doxo-induced Caspase 3/7 activities were positively correlated with the p-AKT levels, which was most obvious in HL-7702 cells with similar p-AKT levels to the Bel-7402 cells (Figure 7G). These results suggested that Doxo-induced apoptosis is more likely to occur in HCC cells with high levels of Madcam1, p-AKT and protein translation.

To further support the finding that p-AKT up-regulation in hepatocytes is capable of inducing Doxo sensitivity, we investigated whether p-AKT down-regulation in HCC cells has the opposite effect. To address this, we consequently knocked down Madcam1 and AKT, followed by Wortmannin treatment in Bel-7402 cells, and found that the p-AKT level was gradually decreased (Figure 7H-7I) and accompanied by gradually decreased Doxo-induced Caspase 3/7 activities (Figure 7J), suggesting that a reduction of p-AKT may decrease Doxo sensitivity in HCC cells.

## DISCUSSION

One purpose of the present study is to identify potential Doxo target(s) to enhance efficacy of Doxo in HCC treatment. The most ideal target(s) are cell membrane-bound proteins. Although Madcam1 was reported to bind integrin α4β7 and mediate lymphocyte adhesion to vascular surfaces [35], no direct evidence has indicated that Madcam1 is localized to the membrane. In HCC cells, we found that Madcam1 was primarily located in the cytoplasm. Interestingly, the subcellular localization of Madcam1 overlapped with the ER. We have described that Madcam1 is associated with protein translation initiation. Thus, we propose that Madcam1 may have another function in regulating protein synthesis in HCC cells.

Many publications have documented that the overexpression of eIF4E may contribute to tumorigenesis by inhibiting apoptosis, while enhanced expression or activity of 4EBP promotes cell death [36-37]. Thus, we suggest that the Doxo-induced pro-apoptotic phenotype in HCC cells may result, in part, from the blockage of eIF4E-mediated protein translation initiation because Doxo prevents eIF4E release from the eIF4E-4EBP1 complexes. Although Doxo-induced Madcam1 down-regulation is also dependent on reduced eIF4E-mediated translation, Madcam1 is more sensitive to Doxo. Because Madcam1 was the most unstable protein among the proteins tested, this suggests that Madcam1 turnover is much more rapid than other proteins and it may have a more rapid response to Doxo treatment. Therefore, Madcam1 down-regulation may be one of the first events to occur after Doxo begins to suppress protein translation, and it acts as to initiate the Doxo-induced pro-apoptotic cascade.

Doxo has attracted attention for use in combined therapies with inhibitors against signaling pathways or proteins that are important for tumor cell growth and survival [38-41]. Because the Madcam1 protein can be easily down-regulated by Doxo and functions to stimulate anti-apoptotic AKT signaling, Madcam1 inhibition not only decreases the expression of a protein that is important during tumorigenesis but also blocks signaling that may induce drug resistance. Therefore, Madcam1 inhibition may sensitize cells to Doxo in HCC treatment. Madcam1 plays a critical role in leukocyte homing to the mucosal immune compartment [6, 42]. Because leukocyte homing is required for the generation and maintenance of immune responses against pathogens, we speculate that suppression of Madcam1 by a specific inhibitor may lead to an impaired immune response to infection. Therefore, a combined treatment that suppresses Madcam1 and enhances immunity at the same time may be a better therapeutic strategy to treat HCC.

Taken together, our study uncovers another mechanism by which Doxo induces apoptosis in HCC cells. We provide a strong rationale for investigating a potential Doxo target that may increase the efficacy of Doxo in HCC treatment. We also demonstrated a positive auto-regulatory loop between the anti-apoptotic Madcam1 protein and PI3K/AKT signaling in the maintenance of protein homeostasis in HCC cells (Figure 7K). Thus, our findings may provide new insights into the treatment of HCC in the future.

## MATERIALS AND METHODS

### Cell culture and vectors

The HCC cell lines Bel-7402 and SMMC-7721 and Hepatocyte line HL-7702 cells were cultured in DMEM. The cells were treated with Doxorubicin (Doxo, 0.5-2.0 μg/ml, Sigma, St. Louis, MO, USA), Actinomycin D (10 μg/ml, Beyotime, Haimen, China), MG132 (25 μM, Cayman, Ann Arbor, MI, USA), Wortmannin (50 μM, Cayman) or cycloheximide (CHX, 50 μg/ml, Sigma) for 2-24 h before harvest. shRNAs against Madcam1 (sh#1 and sh#2) and AKT (sh#1 and sh#2) were purchased from Genechem Biotech LTD (Shanghai, China). The cDNA fragments encoding human Madcam1 were purchased from Origene (Beijing, China) and subcloned into the pcDNA3.1 (+) vector with a C-terminal FLAG tag or into the pLJM-based lentiviral vector with a C-terminal Myc tag. The AKT-Myc expression plasmid was described in our previous study [23]. The primers used for plasmids construction are listed in Supplementary Table.1.

### Immunohistochemistry (IHC), immunofluorescence (IF), and western blotting (WB)

For IHC, the human liver cancer tissue microarray (TMA) slides were purchased from U.S. Biomax (Rockville, MD, USA). Following deparaffinization and rehydration of the tissue sections, antigen retrieval was performed at 100 °C for 2 h with Tris-EDTA buffer, pH 6.0 (Beyotime). Endogenous peroxidases were blocked with 3% peroxide for 20 minutes, followed by three additional rinses in PBS for 5 min. The sections were then blocked in a buffer containing 5% BSA and 0.1% Triton X-100 and incubated overnight in primary antibodies against p-AKT (Abcam, Hong Kong, China, #76297) or Madcam1 (Epitomics, Burlingame, CA, USA, #5490). The signals were detected with the Vectastain ABC kit (Vector Labs, Burlingame, CA, USA). The sections were scored using a semiquantitative scale for each individual tumor tissue on the array slide, with + for weak staining (i.e., 20-40% of cells showing weak to intermediate intensity staining), ++ for strong staining (i.e., > or = 10% of cells showing very intense staining or > 50% of cells showing weak to moderately intense staining, in an appropriate subcellular distribution), +++ very strong staining (i.e., > or = 30% of cells showing very intense staining or > 80% of cells showing moderately intense staining). The scoring results were simplified into the +, ++, or +++ categories. Statistical analysis was performed using the χ^2^ analysis, and a *p*-value of < 0.05 was considered statistically significant.

For IF, the cells were fixed with 4% paraformaldehyde (PFA) for 15 minutes, washed with PBS and blocking buffer (3% FBS+1% HISS+0.1% Triton X-100), and then incubated overnight at 4°C in primary antibodies against Madcam1 (Santa Cruz Biotechnology, Santa Cruz, CA, USA, #sc-365934), cleaved Caspase 3 (Cell signaling technology (CST), Boston, MA, USA, #9664), LC3B (CST, #3868), COXIV (CST, #4850), Calnexin (CST, #2679) or NUP98 (CST, #2598). Fluorescent Alexa-Fluor-488 or -555-conjugated secondary antibodies (life technologies, Carlsbad, CA, USA) were used for detection.

For WB, the proteins were resolved on SDS-PAGE gels followed by standard WB. The primary antibodies used were: FLAG-tag (Sigma, #F3165 or CST, #2368), Myc-tag (CST, #2278 or #2276), GAPDH (CST, #5174), cleaved caspase substrate motif (CST, #8698), CD44 (Epitomics, #1998), CD50 (Epitomics, #3480), CD74 (Epitomics, #2905), CD138 (Epitomics, #5608), MCAM (Epitomics, #2505), CD151 (Epitomics, #5901), CD166 (Epitomics, #3133), CD206 (Epitomics, #5307), RAGE (Abcam, #ab37647), Madcam1 (Epitomics, #5490), GFP (Epitomics, #1533), Fas (Epitomics, #5709), Bax (Epitomics, #1063), p73 (Epitomics, #1636), Livin (CST, #5471), Bcl-2 (Epitomics, #1017), Survivin (CST, #2808), p-eIF2α (CST, #3398), p-4EBP1 (Santa Cruz, #sc-12884 or CST, #2855), p-eIF4E (CST, #9741), p-S6 (CST, #4858), eIF4E (Abcam, #ab33766), p-AKT (Thr308) (Abcam, #ab76297) or AKT (Epitomics, #2957). The human Apoptosis Antibody Array-Membrane (43 Targets) (Abcam, #ab134001) was used to detect possible apoptosis proteins that were highly associated with Doxo and Madcam1, and this analysis was performed in strict accordance to the manufacturer's instruction. The signals were recorded on X-ray film using an HRP-based chemiluminescence analysis. The possible signaling nodes that are involved in apoptosis were screened using the PathScan® stress and apoptosis signaling antibody array kit (CST, #12856) according to the manufacturer's instruction.

### Cell proliferation, Caspase3/7 activity, and quantitative RT-PCR (qPCR)

Cell proliferation was measured by an MTT-based proliferation assay, as previously described [43]. Caspase-3/7 activity was determined using the Caspase-Glo 3/7 assay system (Promega, Madison, WI, USA). Quantitative RT-PCR was performed as previously described [43]. The primers used for qPCR were listed in Supplementary Table.1.

### Co-immunoprecipitation (co-IP)

For immunoprecipitation, the Bel-7402 cells were washed with PBS and subsequently lysed in Western/IP lysis buffer (Beyotime). The protein lysates were centrifuged at 14,000 × g for 10 min to pellet the debris. After preclearing for 1 h with 50 μl of protein A/G-Sepharose (Novex, Oslo, Norway), the supernatants were incubated overnight at 4°C with 3 μg of antibodies against eIF4E (Abcam, #ab33766) crosslinked to protein A/G-Sepharose beads. The beads were washed five times with lysis buffer, resuspended in SDS loading buffer, and analyzed by WB analysis with the indicated antibodies.

### RNA-Immunoprecipitation (RNA-IP)

RNA-IP was performed using a kit from Active Motif and immunoprecipitated using an anti-eIF4E antibody (Abcam, #ab33766) (2 μg for each reaction). The RNA was extracted from the beads using TRIZOL reagent (Life Technologies) and reverse-transcribed using a Primescript II-based reverse transcriptase kit (Takara, Dalian, China). The resulting cDNAs were amplified using primers specific for the target mRNAs listed in Supplementary Table. 1.

### Flow cytometry

An Annexin V-FITC early apoptosis detection kit (CST, #6592) was used to identify the apoptotic DMSO- or Doxo-treated Bel-7402 cells within a cell population. Briefly, the cells were collected by centrifugation, washed with ice-cold PBS and resuspended at 10^5^ cells/ml with 1X Annexin V Binding Buffer. Then, 1 μl of the Annexin V-FITC conjugate and 12.5 μl of the Propidium Iodide (PI) solution were added to each cell suspension (96 μl) and incubated 10 min on ice in the dark. Next, the cell suspension was diluted to a final volume of 250 μl/assay with ice cold 1X Annexin V Binding Buffer, and analyzed immediately using a BD FACS Canto™II cell analyzer (BD Biosciences, San Jose, CA, USA).

### Protein ligation assay

The protein ligation assay (PLA) was performed to identify the interaction between Madcam1 and AKT using the Duolink™ in situ red starter kit (mouse/rabbit) (Sigma, Uppsala, Sweden). Briefly, SMMC-7721 or Bel-7402 cells were seeded on glass cover slips in 24-well plates and fixed before the start of the experiments. Then, the cells were incubated with blocking buffer (supplied by the manufacturer), followed by the indicated primary antibody solution (supplied by the manufacturer), containing rabbit anti-Myc (CST, #2278), mouse anti-FLAG (CST, #8146), rabbit anti-AKT (Epitomics, #4691) or mouse anti-Madcam1(Santa Cruz Biotechnology, #sc-365934) antibodies, overnight at 4°C. The PLA probe solution (supplied by the manufacturer) was then added into each cell sample in a pre-heated humidified chamber for 1 h at 37°C before the Ligase-Ligase solution (supplied by the manufacturer) was added into each sample for 30 min at 37°C. Finally, the glass cover slips were further incubated with the amplification-polymerase solution (supplied by the manufacturer) for 100 min at 37°C before being dried at room temperature in the dark and subjected into microscopic analysis. If the PLA probes are in close proximity ( < 40 nm), bright fluorescent emissions can be detected under a fluorescence microscope.

### Xenograft mouse experiments

Control and Madcam1-knockdown or Madcam1-overexpressing Bel-7402 cells (5×10^6^ cells) (infected with Madcam1-sh1 or lentiviral Madcam1-Myc expressing plasmids, respectively) were subcutaneously injected into 8-week-old athymic nude mice (Bikai, Shanghai, China). The tumor size was measured 30 days after injection, and the tumor volume was calculated as 0.5 × L × W^2^, with L indicating length and W indicating width.

### Statistical analysis

Tests to examine the differences between groups included the Student's *t* test and χ^2^ test. *p* < 0.05 was regarded as statistically significant.

## SUPPLEMENTARY MATERIAL FIGURES AND TABLES



## References

[1] Burden DA, Osheroff N (1998). Mechanism of action of eukaryotic topoisomerase II and drugs targeted to the enzyme. Biochim Biophys Acta.

[2] Lencioni R, de Baere T, Burrel M, Caridi JG, Lammer J, Malagari K, Martin RC, O'Grady E, Real MI, Vogl TJ, Watkinson A, Geschwind JF (2012). Transcatheter treatment of hepatocellular carcinoma with Doxorubicin-loaded DC Bead (DEBDOX): technical recommendations. Cardiovasc Intervent Radiol.

[3] Shin SW (2009). The current practice of transarterial chemoembolization for the treatment of hepatocellular carcinoma. Korean J Radiol.

[4] Kellogg GE, Scarsdale JN, Fornari FA (1998). Identification and hydropathic characterization of structural features affecting sequence specificity for doxorubicin intercalation into DNA double-stranded polynucleotides. Nucleic Acids Res.

[5] Pommier Y, Leo E, Zhang H, Marchand C (2010). DNA topoisomerases and their poisoning by anticancer and antibacterial drugs. Chem Biol.

[6] Briskin M, Winsor-Hines D, Shyjan A, Cochran N, Bloom S, Wilson J, McEvoy LM, Butcher EC, Kassam N, Mackay CR, Newman W, Ringler DJ (1997). Human mucosal addressin cell adhesion molecule-1 is preferentially expressed in intestinal tract and associated lymphoid tissue. Am J Pathol.

[7] Nummer D, Suri-Payer E, Schmitz-Winnenthal H, Bonertz A, Galindo L, Antolovich D, Koch M, Büchler M, Weitz J, Schirrmacher V, Beckhove P (2007). Role of tumor endothelium in CD4+ CD25+ regulatory T cell infiltration of human pancreatic carcinoma. J Natl Cancer Inst.

[8] Liu YX, Yoshino T, Ohara N, Oka T, Jin ZS, Hayashi K, Akagi T (2001). Loss of expression of alpha4beta7 integrin and L-selectin is associated with high-grade progression of low-grade MALT lymphoma. Mod Pathol.

[9] Takata K, Tanino M, Ennishi D, Tari A, Sato Y, Okada H, Maeda Y, Goto N, Araki H, Harada M, Ando M, Iwamuro M, Tanimoto M, Yamamoto K, Gascoyne RD, Yoshino T (2014). Duodenal follicular lymphoma: comprehensive gene expression analysis with insights into pathogenesis. Cancer Sci.

[10] Zhao N, Wang R, Zhou L, Zhu Y, Gong J, Zhuang SM (2014). MicroRNA-26b suppresses the NF-κB signaling and enhances the chemosensitivity of hepatocellular carcinoma cells by targeting TAK1 and TAB3. Mol Cancer.

[11] Kunter I, Erdal E, Nart D, Yilmaz F, Karademir S, Sagol O, Atabey N (2014). Active form of AKT controls cell proliferation and response to apoptosis in hepatocellular carcinoma. Oncol Rep.

[12] Huo X, Zhang Q, Liu AM, Tang C, Gong Y, Bian J, Luk JM, Xu Z, Chen J (2013). Overexpression of Yes-associated protein confers doxorubicin resistance in hepatocellullar carcinoma. Oncol Rep.

[13] Chen X, Lingala S, Khoobyari S, Nolta J, Zern MA, Wu J (2011). Epithelial mesenchymal transition and hedgehog signaling activation are associated with chemoresistance and invasion of hepatoma subpopulations. J Hepatol.

[14] Wei W, Chua MS, Grepper S, So SK (2011). Soluble Frizzled-7 receptor inhibits Wnt signaling and sensitizes hepatocellular carcinoma cells towards doxorubicin. Mol Cancer.

[15] Zheng T, Wang J, Song X, Meng X, Pan S, Jiang H, Liu L (2010). Nutlin-3 cooperates with doxorubicin to induce apoptosis of human hepatocellular carcinoma cells through p53 or p73 signaling pathways. J Cancer Res Clin Oncol.

[16] Giovannini C, Gramantieri L, Chieco P, Minguzzi M, Lago F, Pianetti S, Ramazzotti E, Marcu KB, Bolondi L (2009). Selective ablation of Notch3 in HCC enhances doxorubicin's death promoting effect by a p53 dependent mechanism. J Hepatol.

[17] Choi J, Yip-Schneider M, Albertin F, Wiesenauer C, Wang Y, Schmidt CM (2008). The effect of doxorubicin on MEK-ERK signaling predicts its efficacy in HCC. J Surg Res.

[18] Manov I, Bashenko Y, Eliaz-Wolkowicz A, Mizrahi M, Liran O, Iancu TC (2007). High-dose acetaminophen inhibits the lethal effect of doxorubicin in HepG2 cells: the role of P-glycoprotein and mitogen-activated protein kinase p44/42 pathway. J Pharmacol Exp Ther.

[19] Cuconati A, Mills C, Goddard C, Zhang X, Yu W, Guo H, Xu X, Block TM (2013). Suppression of AKT anti-apoptotic signaling by a novel drug candidate results in growth arrest and apoptosis of hepatocellular carcinoma cells. PLoS One.

[20] Grabinski N, Ewald F, Hofmann BT, Staufer K, Schumacher U, Nashan B, Jücker M (2012). Combined targeting of AKT and mTOR synergistically inhibits proliferation of hepatocellular carcinoma cells. Mol Cancer.

[21] Schmitz KJ, Wohlschlaeger J, Lang H, Sotiropoulos GC, Malago M, Steveling K, Reis H, Cicinnati VR, Schmid KW, Baba HA (2008). Activation of the ERK and AKT signalling pathway predicts poor prognosis in hepatocellular carcinoma and ERK activation in cancer tissue is associated with hepatitis C virus infection. J Hepatol.

[22] Simioni C, Martelli AM, Cani A, Cetin-Atalay R, McCubrey JA, Capitani S, Neri LM (2013). The AKT inhibitor MK-2206 is cytotoxic in hepatocarcinoma cells displaying hyperphosphorylated AKT-1 and synergizes with conventional chemotherapy. Oncotarget.

[23] Wang J, Lin J, Pan Q, Yu Y, Sun F (2014). Cluster of differentiation 166(CD166) regulated by phosphatidylinositide 3-Kinase (PI3K)/AKT signaling to exert its anti-apoptotic role via yes-associated protein (YAP) in liver cancer. J Biol Chem.

[24] Wang J, Zhang Y, Weng W, Qiao Y, Ma L, Xiao W, Yu Y, Pan Q, Sun F (2013). Impaired phosphorylation and ubiquitination by p70 S6 kinase (p70S6K) and Smad ubiquitination regulatory factor 1 (Smurf1) promote tribbles homolog 2 (TRIB2) stability and carcinogenic property in liver cancer. J Biol Chem.

[25] Wang J, Liu X, Wu H, Ni P, Gu Z, Qiao Y, Chen N, Sun F, Fan Q (2010). CREB up-regulates long non-coding RNA, HULC expression through interaction with microRNA-372 in liver cancer. Nucleic Acids Res.

[26] Scheper GC, Proud CG (2002). Does phosphorylation of the cap-binding protein eIF4E play a role in translation initiation?. Eur J Biochem.

[27] Sonenberg N, Hinnebusch AG (2009). Regulation of translation initiation in eukaryotes: mechanisms and biological targets. Cell.

[28] Dix MM, Simon GM, Cravatt BF (2008). Global mapping of the topography and magnitude of proteolytic events in apoptosis. Cell.

[29] Hansen RK, Parra I, Lemieux P, Oesterreich S, Hilsenbeck SG, Fuqua SA (1999). Hsp27 overexpression inhibits doxorubicin-induced apoptosis in human breast cancer cells. Breast Cancer Res Treat.

[30] Kessler SM, Pokorny J, Zimmer V, Laggai S, Lammert F, Bohle RM, Kiemer AK (2013). IGF2 mRNA binding protein p62/IMP2-2 in hepatocellular carcinoma: antiapoptotic action is independent of IGF2/PI3K signaling. Am J Physiol Gastrointest Liver Physiol.

[31] Kaczmarek A, Krysko O, Heyndrickx L, Løve Aaes T, Delvaeye T, Bachert C, Leybaert L, Vandenabeele P, Krysko DV (2013). TNF/TNF-R1 pathway is involved in doxorubicin-induced acute sterile inflammation. Cell Death Dis.

[32] Sonenberg N, Gingras AC (1998). The mRNA 5′ cap-binding protein eIF4E and control of cell growth. Curr Opin Cell Biol.

[33] Reid DW, Nicchitta CV (2015). Diversity and selectivity in mRNA translation on the endoplasmic reticulum. Nat Rev Mol Cell Biol.

[34] Liu T, Singh R, Rios Z, Bhushan A, Li M, Sheridan PP, Bearden SE, Lai JC, Agbenowu S, Cao S, Daniels CK (2015). Tyrosine phosphorylation of HSC70 and its interaction with RFC mediates methotrexate resistance in murine L1210 leukemia cells. Cancer Lett.

[35] Yu Y, Zhu J, Huang PS, Wang JH, Pullen N, Springer TA (2013). Domain 1 of mucosal addressin cell adhesion molecule has an I1-set fold and a flexible integrin-binding loop. J Biol Chem.

[36] Polunovsky VA, Rosenwald IB, Tan AT, White J, Chiang L, Sonenberg N, Bitterman PB (1996). Translational control of programmed cell death: eukaryotic translation initiation factor 4E blocks apoptosis in growth-factor-restricted fibroblasts with physiologically expressed or deregulated Myc. Mol Cell Biol.

[37] Li S, Takasu T, Perlman DM, Peterson MS, Burrichter D, Avdulov S, Bitterman PB, Polunovsky VA (2003). Translation factor eIF4E rescues cells from Myc-dependent apoptosis by inhibiting cytochrome c release. J Biol Chem.

[38] Liu F, Wang P, Jiang X, Tan G, Qiao H, Jiang H, Krissansen GW, Sun X (2008). Antisense hypoxia-inducible factor 1alpha gene therapy enhances the therapeutic efficacy of doxorubicin to combat hepatocellular carcinoma. Cancer Sci.

[39] Piguet AC, Semela D, Keogh A, Wilkens L, Stroka D, Stoupis C, St-Pierre MV, Dufour JF (2008). Inhibition of mTOR in combination with doxorubicin in an experimental model of hepatocellular carcinoma. J Hepatol.

[40] Huynh H, Chow PK, Soo KC (2007). AZD6244 and doxorubicin induce growth suppression and apoptosis in mouse models of hepatocellular carcinoma. Mol Cancer Ther.

[41] Abou-Alfa GK, Johnson P, Knox JJ, Capanu M, Davidenko I, Lacava J, Leung T, Gansukh B, Saltz LB (2010). Doxorubicin plus sorafenib vs doxorubicin alone in patients with advanced hepatocellular carcinoma: a randomized trial. JAMA.

[42] Berlin C, Berg EL, Briskin MJ, Andrew DP, Kilshaw PJ, Holzmann B, Weissman IL, Hamann A, Butcher EC (1993). Alpha 4 beta 7 integrin mediates lymphocyte binding to the mucosal vascular addressin MAdCAM-1. Cell.

[43] Wang J, Park JS, Wei Y, Rajurkar M, Cotton JL, Fan Q, Lewis BC, Ji H, Mao J (2013). TRIB2 acts downstream of Wnt/TCF in liver cancer cells to regulate YAP and C/EBPα function. Mol Cell.

