# Supplementary material for: Doxorubicin induces apoptosis by targeting Madcam1 and AKT and inhibiting protein translation initiation in hepatocellular carcinoma cells
Source: Oncotarget. 2015 Jun 8;6(27):24075–91. doi: 10.18632/oncotarget.4373 (PMC4695171; doi:10.18632/oncotarget.4373)
Supplement: Supplementary file 1 [file oncotarget-06-24075-s001.pdf]

Doxorubicin induces apoptosis by targeting Madcam1 and AKT and inhibiting protein translation initiation in hepatocellular carcinoma cells

Supplementary Material

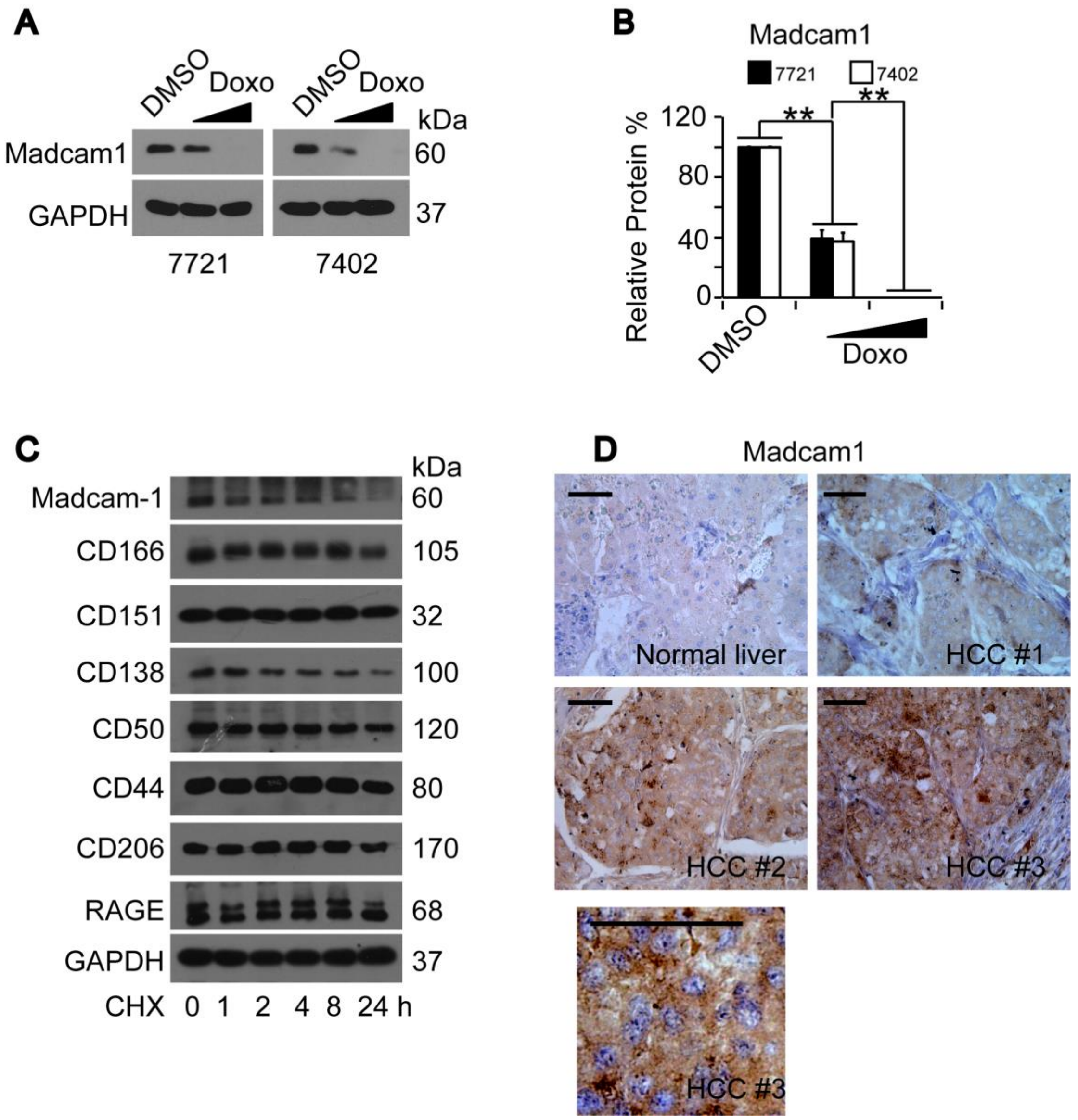

Supplementary Figure 1: Madcam1 expressions in HCC tissues and HCC cells under different treatment

(A-B) Doxorubicin reduced Madcam1 expression. SMMC-7721 and Bel-7402 cells were treated with DMSO and increasing concentration of Doxo (final concentration 0.5-2.0 μg/ml) for 24 h before harvest for WB analysis. The representative images of WB are shown in panel A, and the relative expression

levels of Madcam1 were calculated as the ratio to GAPDH, and the data are shown in the panel B. The “DMSO” group was arbitrarily set to 100 %.

(C) CHX chase analysis of proteins as indicated. Bel-7402 cells were treated with CHX (final concentration 50 μg/ml) for indicated time before harvest for detecting proteins as indicated using WB analysis.

(D) Representative IHC images of Normal liver (n=5) and HCC tissues (n=24) stained using anti-Madcam1 antibodies. Enlarged Image of HCC#3 is shown in the bottom. Scale bar, 150 μM.

Data are shown as Mean + SD from three independent experiments (including WB). **, $p<0.01$ using student t test.

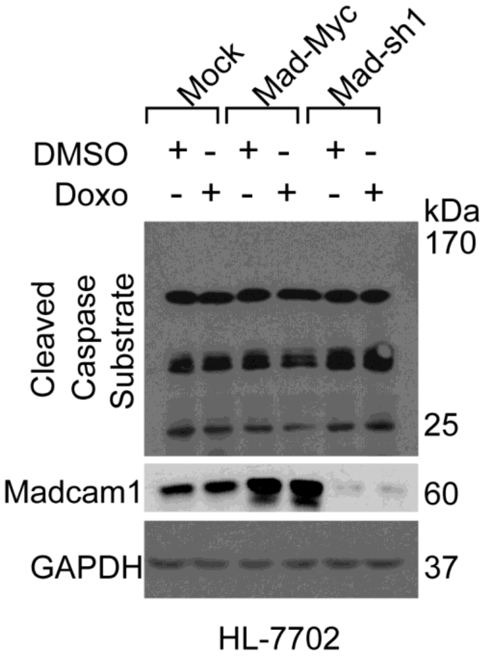

Supplementary Figure 2: Madcam1 had no influence on Doxorubicin-induced effects in hepatocytes

Control (Mock) and HL-7702 hepatocytes with either Madcam1 overexpressed or knocked down were treated with same amount of DMSO or Doxo (final concentration 2.0 μg/ml) for 24 h before harvest for testing cleaved Caspase substrates using anti-cleaved Caspase substrates antibodies by WB.

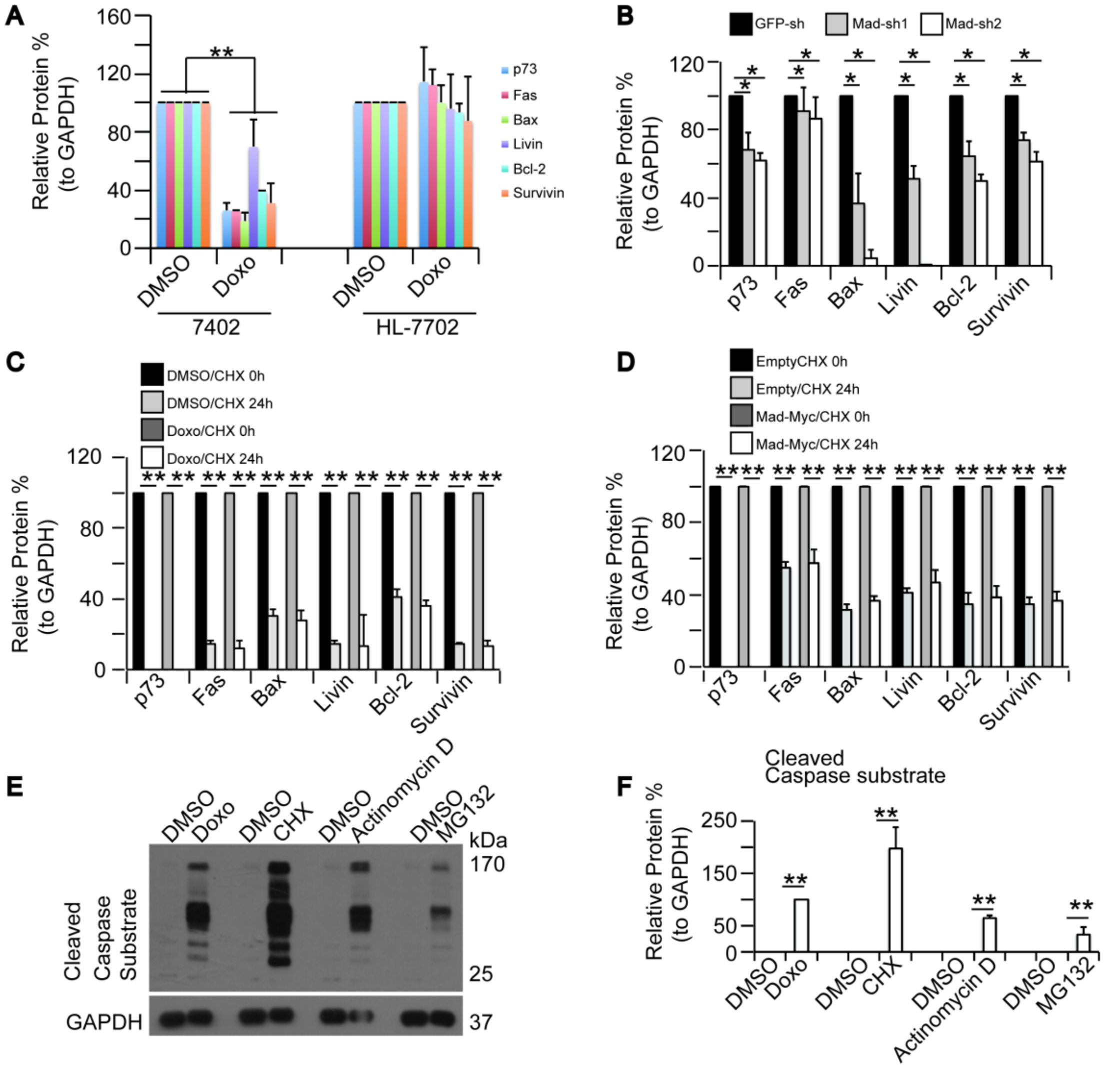

Supplementary Figure 3: Doxorubicin and Madcam1 did not influence protein stability; inhibition of protein synthesis by CHX had the most obvious effects on the induction of apoptosis

(A) Doxo had inhibitory effects on protein expression only in Bel-7402 cells but not in HL-7702 cells. Relative expression levels of protein as indicated in Figure 3E were calculated as the ratio to GAPDH. The data from “DMSO” groups were arbitrarily set to 100 %.

(B) Knockdown of Madcam1 inhibited protein expression. Relative expression levels of proteins as indicated in Figure 3F in Bel-7402 cells were calculated as the ratio to GAPDH. The data from “GFP-sh” groups were arbitrarily set to 100 %.

(C) Doxo did not inhibit protein stability. Relative protein levels as indicated

were normalized to that of GAPDH in Bel-7402 cells under same treatment in Figure 3I. The data from “DMSO/CHX 0h” and “Doxo/CHX 0h” were arbitrarily set to 100 %.

(D) Madcam1 did not stimulate protein stability. Relative protein levels as indicated were normalized to that of GAPDH in Bel-7402 cells under same treatment in Figure 3J. The data from “Empty/CHX 0h” and “Madcam1-Myc/CHX 0h” were arbitrarily set to 100 %.

(E-F) Induced apoptosis by chemical reagents. Apoptosis activities in Bel-7402 cells treated with same amount of DMSO, Doxo (final concentration 2.0 μg/ml), CHX (final concentration 50 μg/ml), Actinomycin D (final concentration 10 μg/ml) or MG132 (final concentration 25 uM) for 24 h were measured by WB using anti-cleaved Caspase substrate antibodies. Representative WB images are shown in the panel E. The relative protein expression of cleaved Caspase substrates were calculated as the ratio to GAPDH, and the data are shown in the panel F. The data from “DMSO” groups were arbitrarily set to 100 %.

Data are shown as Mean + SD from three independent experiments (including WB). *, $p<0.05$ and **, $p<0.01$ using student t test.

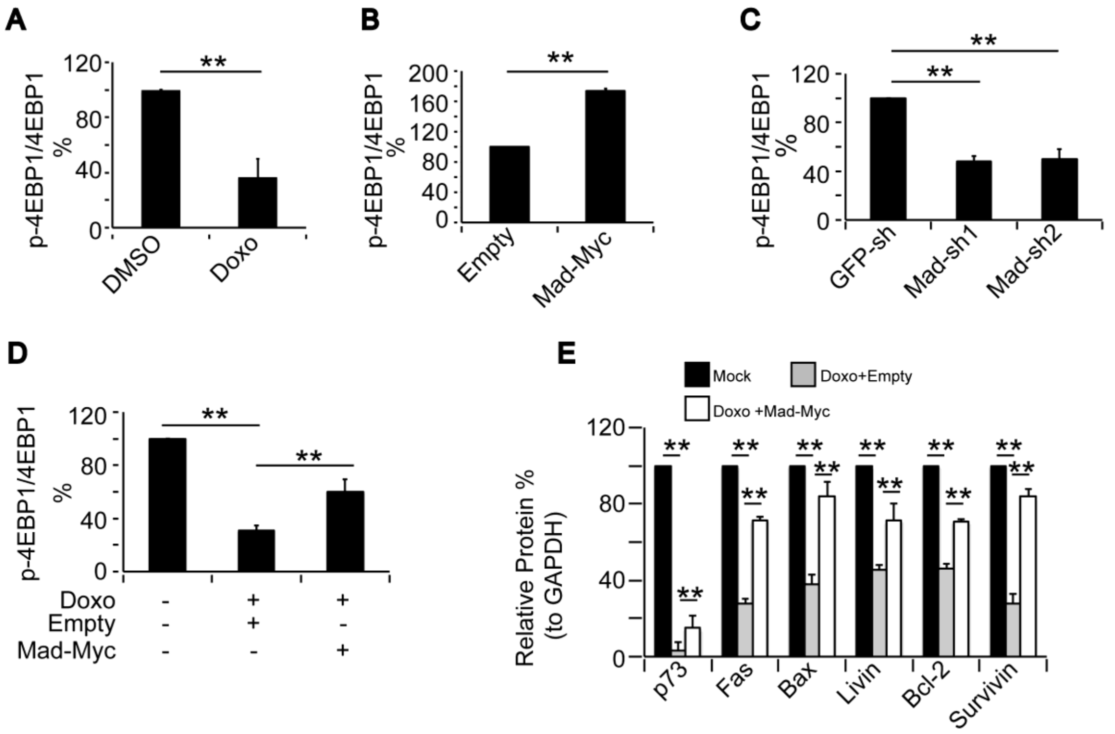

Supplementary Figure 4: Doxorubicin and Madcam1 had opposite effects on the phosphorylation of 4EBP1 and their target protein expression

(A) Doxo reduced phosphorylation of 4EBP1. The treatments to Bel-7402 cells were the same as Figure 4A-4B. Protein levels of the phosphorylated 4EBP1 (p-4EBP1) were normalized to those from total 4EBP1. The data from the "DMSO" group were arbitrarily set to 100%.

(B) Madcam1 overexpression induced phosphorylation of 4EBP1. The treatments to Bel-7402 cells were the same as Figure 4C. Protein levels of the phosphorylated 4EBP1 (p-4EBP1) were normalized to those from total 4EBP1. The data from the "Empty" group were arbitrarily set to 100%.

(C) Madcam1 knockdown reduced phosphorylation of 4EBP1. The treatments to Bel-7402 cells were the same as Figure 4D. Protein levels of the phosphorylated 4EBP1 (p-4EBP1) were normalized to those from total 4EBP1. The data from the "GFP-sh" group was arbitrarily set to 100%.

(D) Madcam1 reversed Doxo-induced de-phosphorylation of 4EBP1. The treatments to Bel-7402 cells were the same as Figure 4H. Protein levels of the phosphorylated 4EBP1 (p-4EBP1) were normalized to those from total 4EBP1. The data from "Mock (untreated)" group were arbitrarily set to 100%.

(E) Madcam1 prevented protein down-regulation by Doxo. Levels of the

indicated proteins in Figure 4I were normalized to those of GAPDH. The data from the “Mock (untreated)” group were arbitrarily set to 100%.
Data are shown as Mean + SD from three independent experiments (including WB). **, $p<0.01$ using student t test.

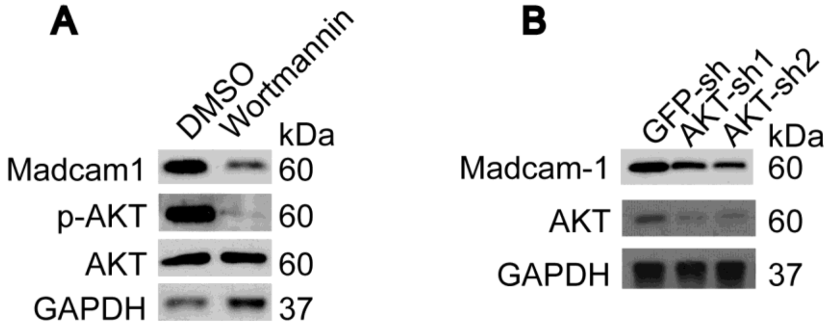

Supplementary Figure 5: Inhibition of AKT reduced Madcam1 expression

(A) Representative Western blots of Madcam1, p-AKT and AKT in Bel-7402 cells treated with same amount of DMSO or Wortmannin (final concentration 50 μM) for 24 h.

(B) Knockdown of AKT reduced Madcam1. Bel-7402 cells infected with GFP-sh, AKT-sh1 or –sh2 were harvested for detecting Madcam1 and AKT by WB.

The data shown above are representative images from three independent experiments.

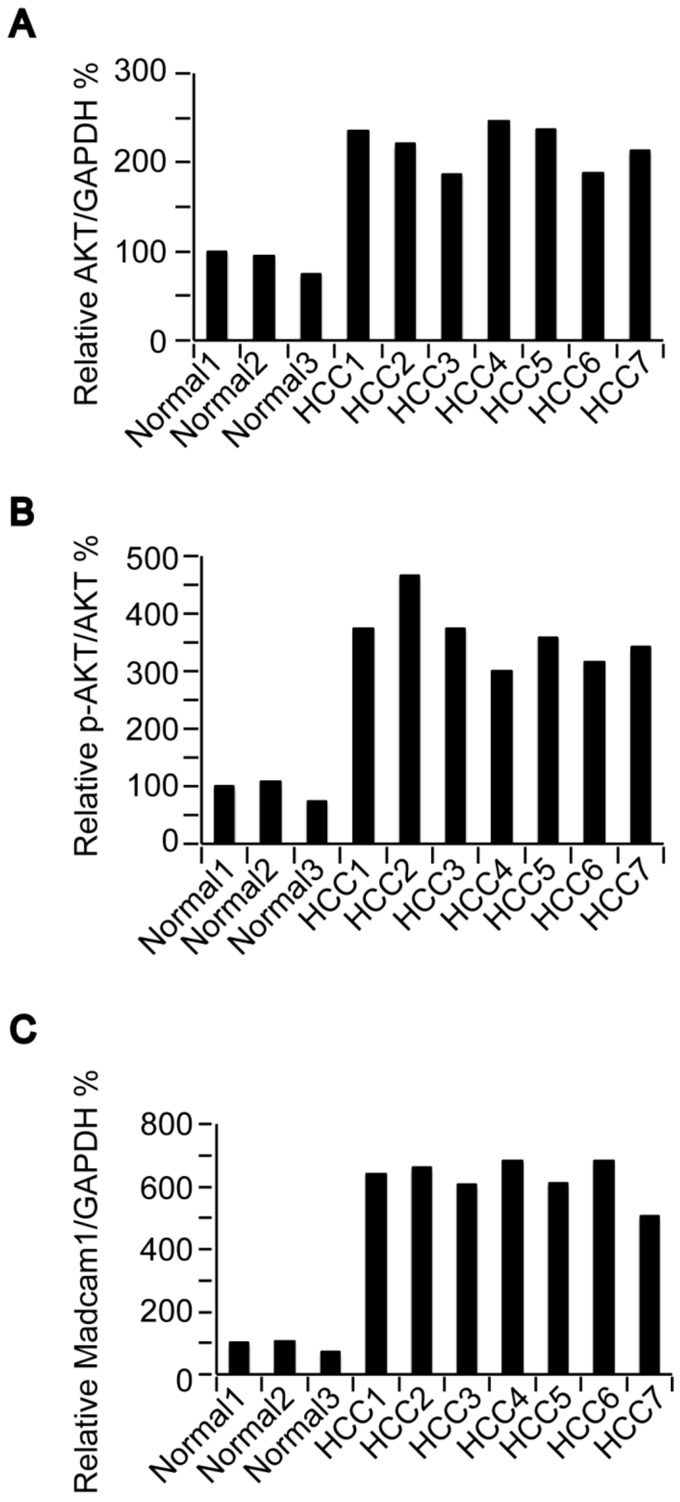

Supplementary Figure 6: p-AKT, AKT and Madcam1 expression in normal liver and HCC tissues

(A) The protein expression levels of AKT from same samples in Figure 7A were normalized to GAPDH. The data from Normal #1 was arbitrarily set to 100%.

(B) The ratios between p-AKT and AKT from same samples in Figure 7A are

shown. The data from Normal #1 was arbitrarily set to 100%.

(C) The protein expression levels of Madcam1 from same samples in Figure 7A were normalized to GAPDH. The data from Normal #1 was arbitrarily set to 100%.

Supplementary Table 1. Primers used in the study.

Supplementary Table 1-1 Primers for qPCR (both for regular-qPCR and RNA-IP-qPCR)

Name	5'-3'
Madcam1-F	GGCCCACGCAGGGAGAAGTGAT
Madcam1-R	GAAGCCTCAGAGAAGCTGGTGG
Fas-F	GTAACTCTACTGTATGTGAAC
Fas-R	TCAGGATTTAAAGTTGGAGAT
Bax-F	CAGTAACATGGAGCTGCAGAG
Bax-R	GGAAGTCCAATGTCCAGCCCA
Bcl2-F	CCTATCAACAACAAGGTTGTT
Bcl2-R	ACTCTTGCAAATTCTACCTTG
p73-F	CCACCTGGACGTACTCCCCGCTC
p73-R	TGAAGTCCCTCCCGAGCTCGTGGTTG
Livin-F	CACCCAGGAGAGAGGTCCAGT
Livin-R	GGGCTCTGCAGATGGGGCACA
Survivn-F	CCTTCTTGGAGGGCTGCGCCT
Survivn-R	CATGGGGTCGTCATCTGGCTC
GAPDH-F	ATCATCCCTGCCTCTACTGG
GAPDH-R	GTCAGGTCCACCACTGACAC

Supplementary Table 1-2 Primers used for construction of protein expression plasmids

Name	5'-3'
Madcam1-FLAG-F(pCDNA3.1+)	GTACGGATCC ATGGATTTCGGACTGGCCCT
Madcam1-FLAG-R(pCDNA3.1+)	CTATCTCGAG TCA CTTGTCATCGTCATCCTTGTAATC GGAGGGGCTGATCCCGACCT
Madcam1-MYC-F (pLJM)	GTACACCGGTATGGATTTCGGACTGGCCCT
Madcam1-MYC-R (pLJM)	GCTAGAATTCTCACAGATCTTCTTCAGAAATAAGTTTTTGTTC GGAGGGGCTGATCCCGACCTG
